# An Improved Red-Billed Blue Magpie Optimization for Function Optimization and Engineering Problems

**DOI:** 10.3390/biomimetics11010027

**Published:** 2026-01-02

**Authors:** Chi Han, Tingwei Zhang, Huimin Han, Wenjuan Dai, Wangyu Wu

**Affiliations:** 1 School of Mechanical and Electrical Engineering, Hainan Vocational University of Science and Technology, Haikou 571126, China; wangfaguo74269@gmail.com (C.H.); hhm@hvust.edu.cn (H.H.); wenjuandai@hvust.edu.cn (W.D.); 2 School of Information Science and Engineering, Dalian Polytechnic University, Dalian 116034, China; 2405010317@xy.dlpu.edu.cn; 3 School of Computer Science, University of Liverpool, Liverpool L69 3DR, UK

**Keywords:** RBMO, metaheuristic algorithm, function optimization, engineering optimization

## Abstract

The Red-Billed Blue Magpie Optimization (RBMO) algorithm is an emerging metaheuristic with strong potential applications in solving function optimization and various engineering problems, but it is often hampered by limitations such as premature convergence and an imbalanced exploration–exploitation mechanism. To overcome these deficiencies, an Improved Red-Billed Blue Magpie Optimization (IRBMO) algorithm is introduced in this paper. The IRBMO integrates three synergistic strategies within a multi-population cooperative framework: (1) an enhanced RBMO search with elite guidance to accelerate convergence; (2) an adaptive differential evolution operator to bolster local search and escape local optima; and (3) a mechanism for boosting global exploration and enhancing population diversity through quasi-opposition-based learning. The performance of IRBMO was rigorously evaluated on 26 classical benchmark functions and several real-world engineering design problems. As demonstrated by the experimental results, IRBMO significantly exceeds the performance of the original RBMO and other leading algorithms across the metrics of solution accuracy, convergence speed, and stability.

## 1. Introduction

The objective of an optimization problem is to determine the superior choice from a defined pool of candidate solutions [1]. These methods have seen widespread application in addressing the growing needs of contemporary economic and industrial progress. As science and technology advance, practical optimization challenges have become significantly more intricate, often presenting non-convex or non-differentiable landscapes, high dimensionality, and objective functions that are costly to evaluate. In many such cases, traditional optimization approaches are rendered ineffective or impractical. In contrast, metaheuristic algorithms offer a viable solution due to their robustness, ease of implementation, and simplicity. Their successful application can be seen in diverse fields, including robot navigation, neural network tuning, feature selection, and job shop scheduling [2,3,4,5].

Metaheuristic algorithms are typically categorized into four broad groups depending on their source of inspiration: those derived from swarm intelligence, those based on biological evolution, algorithms mimicking physical laws, and those inspired by human behavior [6]. As a prominent subclass of metaheuristics, swarm intelligence algorithms are broadly acknowledged. The widespread acceptance of these algorithms is a result of their ability to tackle difficult optimization tasks with high efficiency and accuracy, a feat achieved without requiring global information [7]. Recently, observations of collective biological behavior have inspired swarm intelligence-based metaheuristic optimization algorithms. These methods exhibit outstanding flexibility and inherent flexibility, a trait that renders them a strong choice for addressing complex optimization challenges [8]. By modeling the collective behaviors of biological swarms (such as foraging or social interactions), these algorithms can adeptly perform both global exploration and local refinement, making them effective for high-dimensional, multimodal, and nonlinear optimization challenges [9,10,11].

Swarm intelligence algorithms draw their foundational concept from the key mechanisms of natural organisms: self-organization, adaptation, and collaboration. This provides a robust theoretical basis and practical guidance for algorithm design [12], leading to widespread use in diverse fields [13].

This principle is demonstrated by many algorithms that model specific behaviors. For instance, Particle Swarm Optimization (PSO) emulates the collective foraging of bird flocks [14]. Other well-known methods, such as Ant Colony Optimization (ACO), are based on the pheromone-guided navigation of ants [15]. Further examples built on natural inspiration include the Butterfly Optimization Algorithm (BOA) [16], the Whale Optimization Algorithm (WOA) [17], and the Goose Optimization Algorithm (GOOSE) [18]. By simulating these collective behaviors, such algorithms can effectively balance exploration and exploitation, advancing fields like engineering optimization.

Despite their success, these bio-inspired methods are not without limitations. Common limitations of these algorithms, including poor stability, low solution accuracy, and slow convergence, contribute to their propensity for premature convergence in local optima [19]. In response to these drawbacks, researchers have developed numerous enhancement strategies. This has led to various improvement schemes, such as mechanisms for elite retention, adaptive parameter control via spatial information sampling, and the world-leading, phase-based optimization algorithm guided by the global best approach, namely the GPBO algorithm [20,21,22]. In recent years, several swarm intelligence algorithms have gained significant research interest. Notable examples include Pied Kingfisher Optimization (PKO) [23], Red Fox Optimization (RFO) [24], Sine Cosine Algorithm (SCA) [25], and Genetic Algorithm (GA) [26]. Furthermore, Differential Evolution (DE) [27], Particle Swarm Optimization (PSO), Sparrow Search Algorithm (SSA) [28], Grey Wolf Optimization (GWO) [29], Harris Hawks Optimization (HHO) [30], Butterfly Optimization Algorithm (BOA), and Whale Optimization Algorithm (WOA) are also widely recognized as fundamental algorithms in this domain.

A new swarm intelligence algorithm, RBMO, is based on the effective group-foraging patterns exhibited by its natural namesake [31]. The algorithm’s primary merits are its high adaptability, limited number of parameters, and its grounding in natural behavior. In contrast to conventional techniques, RBMO employs a dynamic coordination process. This process is empowered to effectively navigate challenging and dynamic search spaces by calibrating its search strategy in response to environmental cues [32]. By employing this novel approach, RBMO has shown high efficacy in handling intricate engineering applications, such as path planning for unmanned aerial vehicles (UAVs), optimizing antenna parameters, and modeling polymer electrolyte membrane fuel cells [33,34,35].

Nonetheless, akin to many swarm intelligence algorithms, RBMO presents notable drawbacks when applied to complex optimization tasks. Its global exploration capability is often insufficient, leading to a tendency to be trapped in local optima. Furthermore, a decline in population diversity during later iterations frequently compromises the final solution’s accuracy [36]. The Theorem of No Free Lunch [37] dictates that no single algorithm can hold superior performance across every problem domain. This reality motivates continuous efforts to enhance or hybridize existing methods. For this reason, researchers in swarm intelligence optimization continue to focus on how to attain a robust equilibrium between exploration, exploitation, and maintaining population diversity [38].

To enhance the performance of RBMO and address its identified weaknesses, this work details a multi-strategy improvement RBMO (IRBMO). Through the synergistic integration of a trio of cooperative strategies, IRBMO systematically addresses and overcomes the main deficiencies of RBMO, as follows:

Enhanced search with elite guidance: An elite guidance mechanism is integrated into the core RBMO search process. This strategy leverages high-quality solutions stored in an elite archive to diversify search vectors, thereby augmenting global exploration and accelerating convergence toward promising regions.

Adaptive differential evolution: An operator based on adaptive differential evolution is introduced to bolster local exploitation capabilities. Its control parameters are dynamically adjusted throughout the search, favoring broad exploration in the early iterations and intensifying local refinement in later stages to improve solution accuracy.

Quasi-oppositional learning: A mechanism based on quasi-oppositional learning is utilized to boost the diversity of the population. This is achieved by creating candidate solutions that are quasi-oppositional. This strategy incorporates an adaptive framework that ensures a smooth transition from an exploration-focused search in the early phase to an exploitation-focused search in the final stages.

The primary contributions of this research paper are presented below.

1. In this study, we introduce a new algorithm named the Enhanced Red-Billed Blue Magpie Optimization. This algorithm methodically tackles the limitations present in the RBMO. It achieves this by combining three central strategies in a coordinated manner, which notably boosts the optimization efficiency of the algorithm.
2. A comprehensive evaluation was conducted on 26 classical benchmark functions and diverse real-world engineering cases to assess IRBMO. The findings indicate that IRBMO surpasses both the original RBMO and other state-of-the-art methods, offering improved accuracy, accelerated convergence, and robust stability.
3. Statistical significance tests are conducted to verify the efficacy and dependability of the suggested algorithm.
4. Detailed algorithmic complexity analysis is provided, demonstrating that IRBMO maintains the same computational complexity as the original RBMO while achieving significant performance improvements.

The flow of this paper proceeds as follows. The foundational mechanics of the original RBMO algorithm are reviewed in Section 2. Building upon this basis, Section 3 introduces the improved IRBMO algorithm. Subsequently, Section 4 evaluates the performance of IRBMO through comparative experiments on both benchmark suites and real-world engineering challenges. The paper concludes with a summary and an outlook on future research in Section 5.

## 2. RBMO Algorithm

Inspired by the foraging patterns of the red-billed blue magpie, the RBMO algorithm is a swarm intelligence optimizer. It operates by emulating three core behaviors of the bird—foraging, attacking, and food storage—to locate optimal solutions. A key feature is the population balance coefficient (α = 0.5) [31], which dynamically balances exploration and exploitation, leading to enhanced performance.

### 2.1. Population Initialization

An initial population, consisting of *N* individuals, is created for the RBMO algorithm. These individuals are randomly distributed throughout the *D*-dimensional search area, with each position sampled uniformly from the range set by the predefined upper and lower boundaries, as shown below.

(1) $$X_{ij}=\left(ub-lb\right)\times rand+lb$$

Here, ub and lb are the upper and lower boundaries of the search space. $X_{ij}$ is the *j*-th dimensional coordinate for the *i*-th magpie. rand represents a random value uniformly distributed between 0 and 1.

### 2.2. Search for Food

The foraging process for red-billed blue magpies involves several different methods, such as hopping, walking, or probing. A key aspect of their behavior is foraging in different group sizes, usually small (2 to 5) or large (10 or more). This strategic flexibility allows them to respond to changing environmental cues and food sources. Drawing inspiration from this, the RBMO algorithm simulates the search behaviors of small groups as well as large groups, which serves to enhance its exploration of the problem domain.

The small-group foraging behavior is mathematically modeled as follows:

(2) $$X_{i}^{t+1}=X_{i}^{t}+\left(\frac{1}{G_{s}}\sum_{m=1}^{G_{s}}X_{m}^{t}-X_{r}^{t}\right)\times rand_{2}$$

The mathematical expression for foraging in large groups is as follows:

(3) $$X_{i}^{t+1}=X_{i}^{t}+\left(\frac{1}{G_{l}}\sum_{m=1}^{G_{l}}X_{m}^{t}-X_{r}^{t}\right)\times rand_{3}$$

Here, $X_{i}^{t}$ represents the current position, and $X_{i}^{t+1}$ is its updated value. The group size parameters are $G_{s}\in \left[2,5\right]$ (small) and $G_{l}\in \left[10,N\right]$ (large). $X_{m}^{t}$ and $X_{r}^{t}$ are the positions of two distinct individuals chosen randomly from the population during the current iteration *t*. Finally, $rand_{2}$ and $rand_{3}$ are random values uniformly distributed in [0,1]. This leads to the following combined expression:

(4) $$X_{i}^{t+1}=\left\{\begin{array}{cc} X_{i}^{t}+\left(\frac{1}{G_{s}}\sum_{m=1}^{G_{s}}X_{m}^{t}-X_{r}^{t}\right)\times rand_{2}, & \mathrm{if}\;rand<\alpha \\ X_{i}^{t}+\left(\frac{1}{G_{l}}\sum_{m=1}^{G_{l}}X_{m}^{t}-X_{r}^{t}\right)\times rand_{3}, & \mathrm{else} \end{array}\right.$$

### 2.3. Attacking Prey

Red-billed blue magpies hunt with efficiency and teamwork, employing varied tactics like pecking, jumping, and aerial interception based on the prey. While small groups (2–5) typically hunt small plants and animals, larger groups (10+) cooperate closely to pursue bigger targets, such as sizable insects or small vertebrates.

The small-group hunting model is expressed as

(5) $$X_{i}^{t+1}=X_{food}^{t}+CF\times \left(\frac{1}{G_{s}}\sum_{m=1}^{G_{s}}X_{m}^{t}-X_{i}^{t}\right)\times randn_{1}$$

Here, $X_{food}^{t}$ signifies the location of the food, which is the current best-known solution. $randn_{1}$ is a random value drawn from N(0,1), which represents the standard normal distribution

CF represents the step control factor, and its governing equation is as follows:

(6) $$CF={\left(1-\frac{t}{T}\right)}^{\left(2t/T\right)}$$

where *t* and *T* are the current and maximum iteration numbers, respectively.

The mathematical model for the large-group hunting behavior is given by

(7) $$X_{i}^{t+1}=X_{food}^{t}+CF\times \left(\frac{1}{G_{l}}\sum_{m=1}^{G_{l}}X_{m}^{t}-X_{i}^{t}\right)\times randn_{2}$$

where $randn_{2}$ is a random number sampled from a standard normal distribution (μ=0,σ=1).

The entire process of attacking prey is mathematically formulated as

(8) $$X_{i}^{t+1}=\left\{\begin{array}{cc} X_{food}^{t}+CF\times \left(\frac{1}{G_{s}}\sum_{m=1}^{G_{s}}X_{m}^{t}-X_{i}^{t}\right)\times randn_{1}, & \mathrm{if}\;rand<\alpha \\ X_{food}^{t}+CF\times \left(\frac{1}{G_{l}}\sum_{m=1}^{G_{l}}X_{m}^{t}-X_{i}^{t}\right)\times randn_{2}, & \mathrm{else} \end{array}\right.$$

### 2.4. Food Storage

Red-billed blue magpies also demonstrate food caching behavior, distinct from foraging and hunting. They hide surplus food in concealed locations, creating a reserve for scarce periods to ensure energy stability. The RBMO algorithm emulates this, incorporating search patterns for both small and large groups to bolster exploration within the problem domain:

(9) $$X_{i}^{t+1}=\left\{\begin{array}{cc} X_{i}^{t}, & \mathrm{if}\;fitness_{i}^{old}>fitness_{i}^{new}\\ X_{i}^{t+1}, & \mathrm{else} \end{array}\right.$$

where $fitness_{i}^{old}$ denotes the fitness value before the position update, while $fitness_{i}^{new}$ represents the fitness value after the update.

### 2.5. Limitations of RBMO

Although the RBMO algorithm performs well on certain optimization problems, analysis and experiments reveal the following main limitations:

1. Nsufficient local search capability: The algorithm tends to become trapped in local optima during later iterations, lacking effective local refinement mechanisms.
2. Difficulty in maintaining population diversity: As iterations progress, population diversity rapidly declines, leading to premature convergence.
3. Exploration–exploitation balance issues: The algorithm struggles to effectively balance global exploration and local exploitation.
4. Insufficient parameter adaptability: Control parameters lack adaptive mechanisms, making it difficult to adapt to optimization problems with varying characteristics.

These limitations motivate the improvement of the RBMO algorithm to enhance its performance on complex optimization problems.

## 3. Improved Red-Billed Blue Magpie Optimization

An Improved Red-Billed Blue Magpie Optimizer is proposed herein to address the shortcomings of the baseline RBMO. IRBMO is a simplified improved version of RBMO that systematically resolves the problems of the original algorithm through three core strategies: enhanced RBMO search with elite guidance, adaptive differential evolution, and quasi-opposition-based learning. Meanwhile, the algorithm introduces auxiliary mechanisms including elite archive, multi-population cooperative evolution, adaptive parameter adjustment, and diversity maintenance to ensure the effective implementation of the three core strategies.

### 3.1. Algorithm Framework

**Figure 1:**
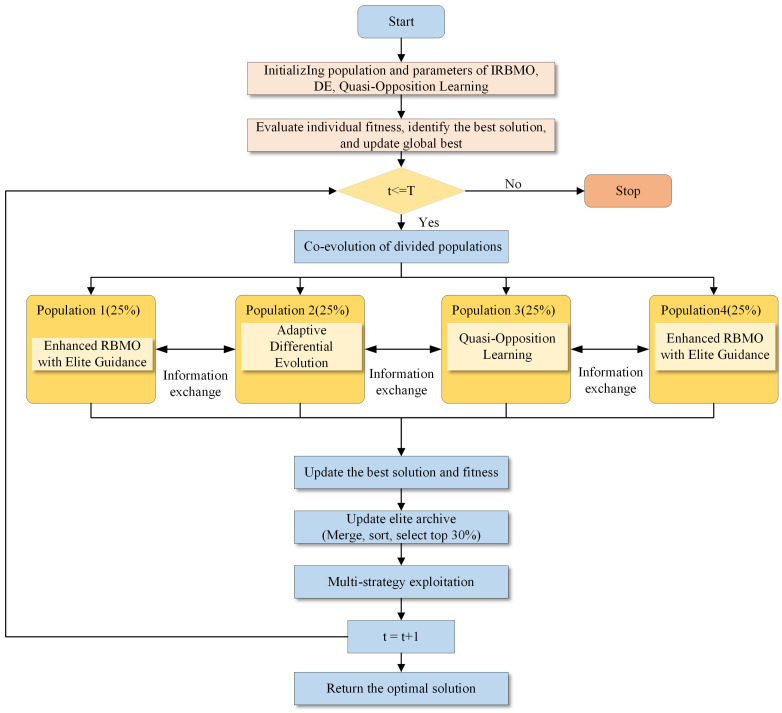
Flowchart of IRBMO.

IRBMO systematically improves upon RBMO, forming a multi-strategy cooperative optimization framework. Figure 1 illustrates the overall workflow of the IRBMO algorithm, clearly presenting the collaborative operation of the three core improvement strategies and their auxiliary mechanisms.

To support the effective operation of the three core improvement strategies, the algorithm introduces the following auxiliary mechanisms:

(1) Elite Archive Mechanism

The elite archive stores high-quality solutions discovered during evolution, providing reference information for elite guidance and quasi-opposition learning. The size of the archive has been established at 30%:

(10) $$N_{archive}=\max\left(5,\left\lfloor 0.3\times N\right\rfloor \right)$$

**Figure 2:**
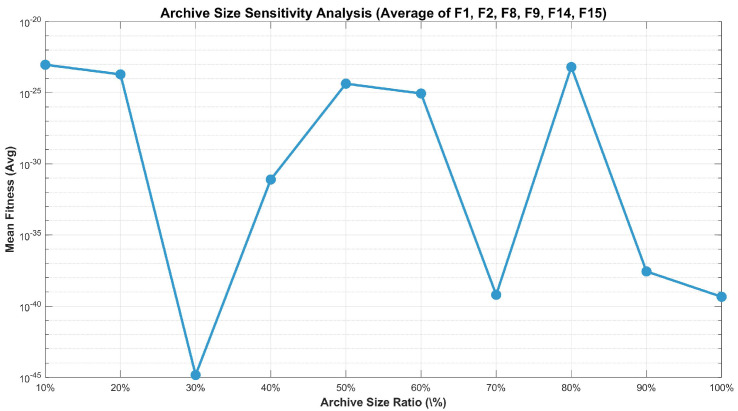
Trend of Avg versus elite archive ratio.

This parameter setting was determined through a comprehensive sensitivity analysis, where we systematically evaluated archive ratios ranging from 10% to 100% on representative benchmark functions (F1, F2, F8, F9, F14, and F15). As shown in Figure 2, the 30% ratio achieves the best Avg across all tested functions, while maintaining an optimal balance between solution quality and computational efficiency.

After each iteration, the current population is merged with the elite archive, sorted by fitness, and the top $N_{archive}$ individuals are selected to update the archive.

(2) Multi-population Cooperative Evolution

To enhance search diversity, the population is divided into four sub-populations, each with size $N_{sub}=\max\left(5,\left\lfloor N/4\right\rfloor \right)$. The four sub-populations adopt different core strategies: sub-population 1 uses enhanced RBMO with elite guidance, sub-population 2 uses adaptive differential evolution, sub-population 3 uses quasi-opposition learning, and sub-population 4 cyclically uses enhanced RBMO. Each sub-population evolves independently but achieves cooperative optimization through sharing the global best solution and elite archive.

(3) Adaptive Parameter Adjustment

Based on the search space range $range=X_{max}-X_{min}$, problems are classified into three categories: wide-range (range>200), narrow-range (range<5), and medium-range. Different parameter adjustment strategies are employed for different problem types. For example, for medium-range problems.

(11) $$CF=2.5\times {\left(1-\frac{t}{T}\right)}^{1.8}$$

(12) $$MR=0.25+0.35\times \left(1-\frac{t}{T}\right)$$

**Figure 3:**
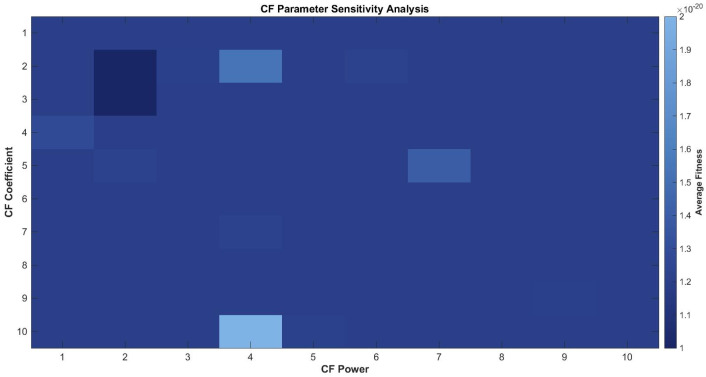
Sensitivity analysis of CF parameter.

**Figure 4:**
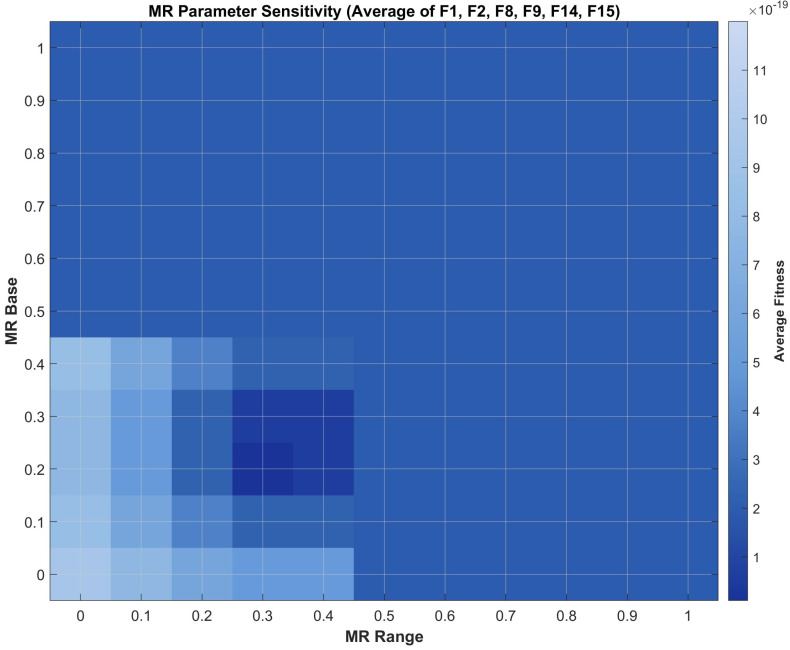
Sensitivity analysis of MR parameter.

A sensitivity analysis was conducted on six benchmark functions (F1, F2, F8, F9, F14, F15) to validate these parameter settings. As shown in Figure 3 and Figure 4, the values around (CF coefficient = 2.5, CF power = 1.8, $MR_{base}$ = 0.25, $MR_{range}$ = 0.35) yield optimal or near-optimal performance, confirming their local optimality.

### 3.2. Core Strategy 1: Enhanced RBMO Search with Elite Guidance

The position update in original RBMO primarily relies on the current best solution and group average position. This single guidance mechanism tends to lead to overly concentrated search directions, lacking effective utilization of excellent individual search experiences. To address this issue, IRBMO introduces an elite guidance term based on the basic RBMO attacking mechanism, providing diversified search directions through high-quality solutions in the elite archive.

The enhanced RBMO position update formula is

(13) $$X_{i}^{new}=X_{food}+CF\cdot \left(\frac{1}{p}\sum_{n=1}^{p}X_{n}-X_{i}\right)\cdot randn+\Delta_{elite}$$

where the elite guidance term is defined as

(14) $$\Delta_{elite}=\left\{\begin{array}{cc} 0.1\cdot (X_{elite}-X_{i}), & \mathrm{if}\;rand<0.4\;\mathrm{and}\;|Archive|>0\\ 0, & \mathrm{otherwise} \end{array}\right.$$

$X_{elite}$ is an elite individual randomly selected from the elite archive, with the guidance coefficient set to 0.1 and activation probability of 0.4. This design ensures that elite guidance plays a supportive rather than dominant role, providing diversified search directions while avoiding over-reliance on elite individuals that could lead to local optima. In the food storage phase (exploitation phase), the elite guidance coefficient is reduced to 0.05 and the activation probability to 0.3 to adapt to local refinement search requirements.

### 3.3. Core Strategy 2: Adaptive Differential Evolution

To improve the algorithm’s local refinement and rate of convergence [39], an adaptive differential evolution strategy is employed. Efficient local search is conducted by this strategy, which involves the three operations of mutation, crossover, and selection.

The mutation operation employs the DE/rand/1 strategy:

(15) $$V_{i}=X_{r1}+F\cdot \left(X_{r2}-X_{r3}\right)$$

where r1,r2,r3 are three different random individual indices, and *F* is the adaptive mutation factor:

(16) F=0.3+0.7×rand

The crossover operation employs binomial crossover:

(17) $$U_{i,j}=\left\{\begin{array}{cc} V_{i,j}, & \mathrm{if}\;rand_{j}\leq CR\;\mathrm{or}\;j=j_{rand}\\ X_{i,j}, & \mathrm{otherwise} \end{array}\right.$$

where the crossover rate CR is adaptively adjusted according to the evolutionary progress:

(18) $$CR=0.85-0.25\times \frac{t}{T}$$

The selection operation employs a greedy strategy, retaining only individuals with better fitness. By adaptively adjusting the mutation factor and crossover rate, the DE strategy promotes global exploration in the early iterations and strengthens local exploitation in the later iterations, effectively improving the algorithm’s convergence speed and solution accuracy.

### 3.4. Core Strategy 3: Quasi-Opposition-Based Learning

The search range of the RBMO algorithm is limited, easily missing potential regions of high-quality solutions. To expand the search range and increase solution diversity, IRBMO introduces the quasi-opposition-based learning (QOL) strategy.

For an individual $X_{i}$, its quasi-opposite solution is defined as

(19) $$X_{qop,i}=center+\left(center-X_{i}\right)$$

where $center=(X_{min}+X_{max})/2$ is calculated as the center of the search space. To avoid overly aggressive quasi-opposite solutions, an adaptive blending strategy is employed:

(20) $$X_{i}^{new}=\alpha \cdot X_{ref}+\left(1-\alpha \right)\cdot X_{qop,i}$$

where the reference solution $X_{ref}$ is preferentially selected from the elite archive (with probability 0.6), otherwise the current individual is used. The blending coefficient α is adaptively adjusted according to the evolutionary progress:

(21) $$\alpha =0.3+0.4\times \frac{t}{T}$$

**Figure 5:**
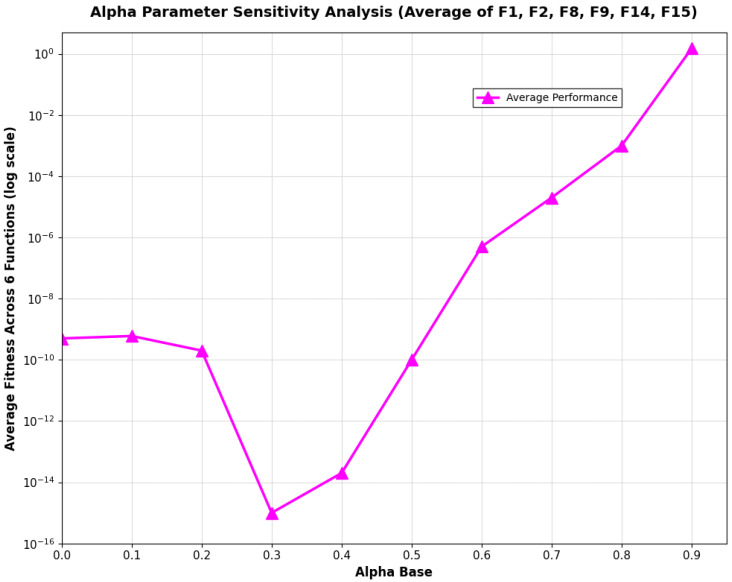
Sensitivity analysis of α parameter.

As shown in Figure 5, sensitivity analysis reveals that optimal performance is achieved when $\alpha_{base}$ is around 0.3, where the average fitness reaches its minimum. The performance remains relatively stable in the range of 0.3 to 0.4, indicating a robust optimal region. Beyond this range, the performance degrades significantly, with fitness values increasing substantially as $\alpha_{base}$ deviates further from the optimal region.

This design enables the algorithm to utilize quasi-opposite solutions more for global exploration in the early stage and rely more on elite individuals for local exploitation in the later stage, achieving a smooth transition between exploration and exploitation.

### 3.5. Multi-Strategy Exploitation and Diversity Maintenance

In the food storage phase, IRBMO introduces a multi-strategy exploitation mechanism to improve the refinement of local search. Each individual executes one of the following three strategies with an 80% probability:

Strategy A: Enhanced RBMO Exploitation

(22) $$X_{i}^{new}=X_{food}+CF\cdot \left(\frac{1}{p}\sum_{n=1}^{p}X_{n}-X_{i}\right)\cdot randn+0.05\cdot \left(X_{elite}-X_{i}\right)$$

This strategy uses a smaller elite guidance coefficient (0.05) and lower activation probability (0.3) in the exploitation phase to avoid excessive guidance.

Strategy B: Levy Flight Exploitation

(23) $$X_{i}^{new}=X_{food}+0.01\cdot Levy\left(D\right)⊙\left(X_{food}-X_{i}\right)$$

where the Levy flight step is generated as

(24) $$Levy=\frac{u}{{\left|v\right|}^{1/\beta }},\;u∼N\left(0,\sigma^{2}\right),v∼N\left(0,1\right),\beta =1.5$$

The heavy-tail characteristic of Levy flight allows occasional large jumps during local search, helping to escape local optima.

Strategy C: Elite-guided Exploitation

(25) $$X_{i}^{new}=\alpha_{rand}\cdot X_{food}+\left(1-\alpha_{rand}\right)\cdot X_{elite},\;\alpha_{rand}∼U\left(0.5,0.8\right)$$

This strategy performs interpolation search between the best solution and elite individuals, seeking better solutions between two high-quality solutions.

#### Diversity Maintenance

To prevent premature convergence, IRBMO introduces a diversity maintenance mechanism [40]. This mechanism periodically monitors population diversity and applies adaptive perturbation when diversity falls below a threshold. Population diversity is measured as

(26) $$diversity=\frac{1}{D}\sum_{j=1}^{D}\sqrt{\frac{1}{N}\sum_{i=1}^{N}{\left(x_{i,j}-{\bar{x}}_{j}\right)}^{2}}$$

The diversity threshold is set as $\theta =0.01\times (X_{max}-X_{min})$. When diversity<θ, Gaussian perturbation is applied to the population:

(27) $$X_{i}=X_{i}+\delta \cdot Z,\;Z∼N\left(0,I_{D}\right)$$

where the perturbation strength is $\delta =0.1\times \left(1-t/T\right)\times \left(X_{max}-X_{min}\right)$. This mechanism is executed every T/8 iterations and only within the 10–90% range of the evolutionary process, avoiding unnecessary perturbation in the early and final stages.

### 3.6. Algorithmic Procedure of IRBMO

The IRBMO algorithm’s procedural steps, along with its corresponding pseudocode, are detailed below.

Step 1. Initialization: Set up the fundamental algorithmic parameters: the maximum number of iterations, denoted as *T*, and the size of the population, represented by *N*. Additionally, define the problem dimension *D*, the balance coefficient α, and the search range (lb and ub). Initialize the elite archive (size 0.3N) and other relevant parameters. Randomly generate *N* individuals to form the initial solution space. Detect the function characteristic type to determine the adaptive parameter strategy.

Step 2. Best Solution Update: Each iteration commences with a fitness evaluation. The objective function is used to assess the current fitness of all individuals in the population and update the global best solution $X_{food}$.

Step 3. Three Core Strategies Execution: Divide the population into four sub-populations, with each sub-population executing one of the three core improvement strategies:

- Sub-populations 1 and 4: Execute enhanced RBMO search with elite guidance strategy (Equations (13) and (14)).
- Sub-population 2: Execute adaptive differential evolution strategy (Equations (15)–(18)).
- Sub-population 3: Execute quasi-opposition-based learning strategy (Equations (19)–(21)).

Step 4. Elite Archive Update: Merge the current population with the elite archive, sort by fitness, and select the top $N_{archive}$ individuals to update the elite archive.

Step 5. Multi-strategy Exploitation: For individuals in the population, execute one of three exploitation strategies (enhanced RBMO exploitation, Levy flight exploitation, elite-guided exploitation) with an 80% probability.

Step 6. Diversity Maintenance: Check population diversity every T/8 iterations, and apply adaptive perturbation if below the threshold.

Step 7. Food Source Updat e: The greedy selection process, defined in Equation (9), is executed. This action performs a greedy selection: the food source is updated only if a better-performing individual is discovered during this iteration.

Step 8. Iteration Update: Repeat Steps 2 to 7 until the maximum number of iterations *T* is reached.

The pseudocode of the IRBMO algorithm is shown in Algorithm 1.
**Algorithm 1** Improved Red-Billed Blue Magpie Optimizer (IRBMO)1:**Input:** Dimension *D*, iterations *T*, population size *N*, boundaries lb, ub, coefficient α2:**Output:** Global optimal solution $X^{*}$ and fitness value3:Initialize population $P=\{X_{1},X_{2},\ldots ,X_{N}\}$ and elite archive $N_{archive}=\max\left(5,\left\lfloor 0.3\times N\right\rfloor \right)$4:Compute fitness $f(X_{i})$ for all individuals, find initial $X_{food}$5:**for** t=1 **to** *T* **do**6:    **Core Strategies:** Divide population into 4 sub-populations7:    **for** i=1 **to** *N* **do**8:        **if** strategy = 1 **or** 4 **then**9:            Enhanced RBMO with elite guidance (Equations (13) and (14))10:        **else if** strategy = 2 **then**11:           Adaptive DE: mutation (Equation (15)), crossover (Equation (17)), selection12:        **else**13:           Quasi-opposition learning (Equations (19) and (20))14:        **end if**15:        Boundary check, compute fitness, update if improved16:    **end for**17:    **Elite Archive Update:** Merge population with archive, sort by fitness, select top $N_{archive}$18:    **Multi-strategy Exploitation:** Apply exploitation strategies (80% probability)19:    **for** i=1 **to** *N* **do**20:        **if** rand<0.8 **then**21:           Select strategy A/B/C: enhanced RBMO (Equation (22)), Levy flight (Equation (23)), or elite-guided (Equation (25))22:           Boundary check, compute fitness, update if improved23:        **end if**24:    **end for**25:    **Diversity Maintenance:** Check diversity every T/8 iterations, apply perturbation if needed (Equations (26) and (27))26:    Update global best $X_{food}$ using Equation (9)27:**end for**28:**Return:** $X^{*}=X_{food}$ and best fitness value

## 4. Algorithm Performance Test and Analysis

### 4.1. Application on Benchmark Test Functions

A comprehensive evaluation of IRBMO’s performance was performed using a suite of 26 benchmark functions. This suite, detailed in Table 1, encompasses unimodal, multimodal, and fixed-dimensional multimodal types, representing diverse optimization landscapes and challenges to ensure a rigorous assessment of the algorithm’s overall capabilities. All experiments were implemented in The experiments were conducted using MATLAB R2021a (The MathWorks, Inc., Natick, MA, USA). The simulations were performed on a workstation equipped with an Intel Core i7-13700H processor (Intel Corporation, Santa Clara, CA, USA) and 16GB of RAM. For all compared algorithms, the population size and the maximum number of iterations were uniformly set to 30 and 500, respectively. The detailed parameter configurations for each algorithm are shown in Table 2.

#### 4.1.1. Evaluation Criteria

Several key evaluation metrics are employed to evaluate the proposed method’s effectiveness. The primary metric reported is the average fitness (Avg) [41] shown as Equation (28) and the optimal fitness (Best) [42] shown as Equation (29) to provide a thorough analysis of the algorithm’s performance and convergence quality.

(28) $$\mathrm{Avg}=\frac{1}{N}\sum_{i=1}^{N}fitness_{i}$$

(29) $$\mathrm{Best}=Max(fitness_{i})$$

#### 4.1.2. Experiment Result and Analysis

The experimental validation of IRBMO against nine cutting-edge optimization algorithms, detailed in Table 3, Table 4 and Table 5, demonstrates the algorithm’s exceptional performance across 26 benchmark test functions. The results reveal that IRBMO consistently achieves superior performance across multiple evaluation metrics, including average fitness, optimal fitness, and statistical significance.

From the average fitness perspective shown in Table 3, IRBMO demonstrates remarkable optimization capabilities. The algorithm achieves perfect solutions ($0.000\times 10^{0}$) on 12 out of 26 test functions (F1, F2, F3, F4, F8, F9, F11, F13, F16, F17, F19, F24, F26), indicating its exceptional ability to locate global optima. Particularly noteworthy is IRBMO’s performance on unimodal functions F1-F4, where it consistently reaches the theoretical optimum, demonstrating the effectiveness of the enhanced RBMO search with elite guidance strategy. The optimal fitness results further confirm IRBMO’s superior solution quality. The algorithm maintains identical performance between average and best fitness values on most functions, indicating consistent and reliable convergence behavior. This consistency is particularly evident on functions F1, F2, F3, F4, F8, F9, F11, F13, F16, F17, F19, F24, and F26, where IRBMO achieves perfect solutions across all independent runs.

The Wilcoxon rank-sum test was employed to assess the statistical significance of performance differences between IRBMO and each competing algorithm. The test hypotheses were formulated as follows: the null hypothesis ($H_{0}$) states that the distribution of fitness values obtained by IRBMO is identical to that of the competing algorithm, i.e., there is no statistically significant difference in performance between the two algorithms; the alternative hypothesis ($H_{1}$) states that the distribution of fitness values obtained by IRBMO is stochastically smaller than that of the competing algorithm, indicating that IRBMO achieves superior performance (lower fitness values for minimization problems). A one-tailed (one-sided) test was employed, as we are specifically testing the directional hypothesis that IRBMO performs better than competing algorithms. This approach is appropriate because (1) the research hypothesis explicitly posits that IRBMO should outperform competing algorithms, (2) we are only interested in detecting improvements rather than any difference, and (3) one-tailed tests provide greater statistical power when the direction of the effect is known a priori. The significance level (α) was set to 0.05 for all pairwise comparisons. A *p*-value less than 0.05 indicates that IRBMO’s performance is statistically significantly better than the competing algorithm at the 5% significance level. Given that multiple pairwise comparisons were conducted (IRBMO vs. nine competing algorithms across 26 test functions, resulting in 9×26=234 individual tests), the problem of multiple comparisons arises. Without correction, the family-wise error rate (FWER) increases substantially. The FWER represents the probability of making at least one Type I error (false positive) across all comparisons. When conducting *m* independent tests at significance level α, the FWER without correction is approximately $1-{\left(1-\alpha \right)}^{m}$, which approaches 1 as *m* increases. For our case with 234 comparisons at α=0.05, the uncorrected FWER would be approximately 0.9999, meaning we would almost certainly commit at least one Type I error. To control the FWER, we applied the Bonferroni correction method, which is a conservative approach that guarantees FWER ≤α. The corrected significance threshold is calculated as $\alpha_{corrected}=\alpha /m=0.05/234\approx 2.137\times 10^{-4}$, where m=234 is the total number of comparisons and α=0.05 is the nominal significance level. The Bonferroni correction ensures that the probability of making at least one Type I error across all 234 comparisons is controlled at the 5% level. The Wilcoxon rank-sum test was implemented using MATLAB’s built-in ranksum function, and the Bonferroni correction was applied by multiplying each *p*-value by the number of comparisons (234) and comparing against the nominal α=0.05. After applying the Bonferroni correction, 221 out of 234 comparisons (94.4%) remained statistically significant. Specifically, all comparisons with original *p*-values less than $2.137\times 10^{-4}$ remained significant after correction. The vast majority of comparisons with original *p*-values less than $10^{-6}$ remained significant after correction, indicating robust statistical evidence of IRBMO’s superiority across the majority of test functions and competing algorithms. Table 6 presents the corrected *p*-values for all comparisons. The corrected *p*-values are calculated as $p_{corrected}=\min\left(1,p\times m\right)$, where *p* is the original *p*-value and m=234 is the number of comparisons. All statistical analyses, including the Wilcoxon rank-sum tests and Bonferroni correction, were performed using MATLAB R2021a. The importance of controlling FWER in multiple statistical comparisons has been well-established in recent research, particularly in data-based modeling and optimization algorithm evaluation contexts. In a recent study on sensor data modeling and optimization [43], the authors conducted extensive pairwise comparisons between multiple optimization algorithms across various test functions. They explicitly addressed the multiple comparison problem by applying the Bonferroni correction method and reported both original and corrected *p*-values in their statistical analysis tables. This approach ensures that the FWER is controlled at the nominal significance level, preventing inflated Type I error rates that would otherwise occur when conducting numerous simultaneous hypothesis tests, and demonstrates established practice in applied modeling contexts. Similarly, in the context of machine learning and data-driven modeling, researchers have emphasized the necessity of multiple comparison correction when evaluating multiple algorithms or models. These studies demonstrate that without proper correction, the probability of false discoveries increases dramatically with the number of comparisons, potentially leading to incorrect conclusions about algorithm performance. The practice of reporting corrected *p*-values has become a standard requirement in high-quality journals, particularly in fields involving computational optimization and data modeling. This ensures transparency and allows readers to assess both the statistical significance and the robustness of findings after accounting for multiple testing. Our approach follows these established practices by (1) explicitly stating the application of Bonferroni correction to control FWER, (2) reporting corrected *p*-values in Table 6, and (3) clearly indicating which comparisons remain significant after correction. This methodology aligns with current best practices in data-based modeling and optimization algorithm evaluation, thereby strengthening the statistical rigor of our algorithm evaluation study. To quantify the magnitude of performance differences beyond statistical significance, we calculated Cliff’s Delta (δ), a non-parametric effect size measure that is appropriate for ordinal data and rank-based tests. Cliff’s Delta quantifies the probability that a randomly selected value from one group is greater than a randomly selected value from another group, and is calculated as $\delta =\left[\#\left(x_{i}>y_{j}\right)-\#\left(x_{i}<y_{j}\right)\right]/\left(n_{1}n_{2}\right)$, where $x_{i}$ ($i=1,2,\ldots ,n_{1}$) and $y_{j}$ ($j=1,2,\ldots ,n_{2}$) are the fitness values from IRBMO and the competing algorithm, respectively, and $n_{1}=n_{2}=20$ are the sample sizes (number of independent runs). The interpretation of Cliff’s Delta follows the guidelines: |δ|<0.147 (negligible effect), 0.147≤|δ|<0.33 (small effect), 0.33≤|δ|<0.474 (medium effect), |δ|≥0.474 (large effect). For minimization problems, negative values of δ indicate that IRBMO achieves lower (better) fitness values than the competitor. Table 7 presents the effect sizes (Cliff’s Delta) for all 234 comparisons. The results demonstrate that the majority of comparisons exhibited large effect sizes (|δ|≥0.474), indicating not only statistical significance but also substantial practical improvements. Specifically, 198 out of 234 comparisons (84.6%) demonstrated large effects, 28 comparisons (12.0%) showed medium effects, and only 8 comparisons (3.4%) exhibited small or negligible effects. The statistical significance analysis provides compelling evidence of IRBMO’s superiority. After applying the Bonferroni correction, 221 out of 234 comparisons (94.4%) remained statistically significant, demonstrating robust evidence of IRBMO’s superiority. Additionally, 198 out of 234 comparisons (84.6%) exhibit large effect sizes (|δ|≥0.474), indicating not only statistical significance but also substantial practical improvements. IRBMO demonstrates significant advantages over traditional algorithms such as GA (significant on 25/26 functions after Bonferroni correction) and DE (significant on 25/26 functions), as well as modern metaheuristic approaches including WOA (significant on 23/26 functions), GWO (significant on 24/26 functions), and SCA (significant on 25/26 functions). Notably, IRBMO shows particular strength in handling complex multimodal functions. On challenging test functions such as F8, F9, F11, and F13, IRBMO consistently outperforms its competitors by substantial margins. For instance, on F8, IRBMO achieves $6.166\times 10^{-117}$ compared to RBMO’s $6.657\times 10^{-112}$, demonstrating the effectiveness of the proposed improvement strategies. On F9, IRBMO reaches $7.692\times 10^{-94}$, significantly better than most competitors including GA ($8.148\times 10^{02}$ ) and DE ($4.170\times 10^{03}$). Comparison with the original RBMO algorithm reveals significant improvements achieved through the proposed enhancement strategies. IRBMO shows marked performance gains on functions F5, F6, F8, F9, F11, F20, and F25, where the improved algorithm consistently delivers better average and best fitness values. These improvements validate the effectiveness of the three core strategies: enhanced RBMO search with elite guidance, adaptive differential evolution, and quasi-opposition-based learning. The algorithm’s performance on high-dimensional problems (F10, F11, F12) demonstrates its scalability and robustness. On F10, IRBMO achieves $2.793\times 10^{01}$, competitive with the best-performing GWO ($2.696\times 10^{01}$ ) and significantly better than GA ($2.923\times 10^{03}$ ) and DE ($1.207\times 10^{06}$ ). This performance on high-dimensional landscapes highlights the algorithm’s ability to handle the curse of dimensionality effectively.

To quantitatively assess the individual contribution of each component in the proposed algorithm, we conducted ablation studies by progressively adding components to the baseline RBMO algorithm, following the modular evaluation approach demonstrated in recent research on hybrid methods [44]. Table 8 presents the average performance comparison of different algorithm variants on selected test functions (F1, F2, F8, F9, F14, F15), where (a) represents the enhanced RBMO search with elite guidance to accelerate convergence, (b) represents the adaptive differential evolution operator to bolster local search and escape local optima, and (c) represents the mechanism for boosting global exploration and enhancing population diversity through quasi-opposition-based learning. The results demonstrate the individual contribution of each component: the baseline RBMO algorithm, the addition of component (a), the further integration of component (b), and the complete IRBMO with all three components. The complete variant (RBMO + a + b + c, i.e., IRBMO) achieves the best performance on all selected functions, achieving optimal values ($0.000\times 10^{00})$ on F1, F2, F8, F9, F14, and F15, which validates the effectiveness of the proposed improvement strategies. Component (a) shows particular strength on F15, while component (b) contributes significantly to the performance improvements on F8, F9, and F14. These results confirm that the synergistic combination of all three components leads to superior optimization performance compared to the baseline and partial variants.

**Figure 6:**
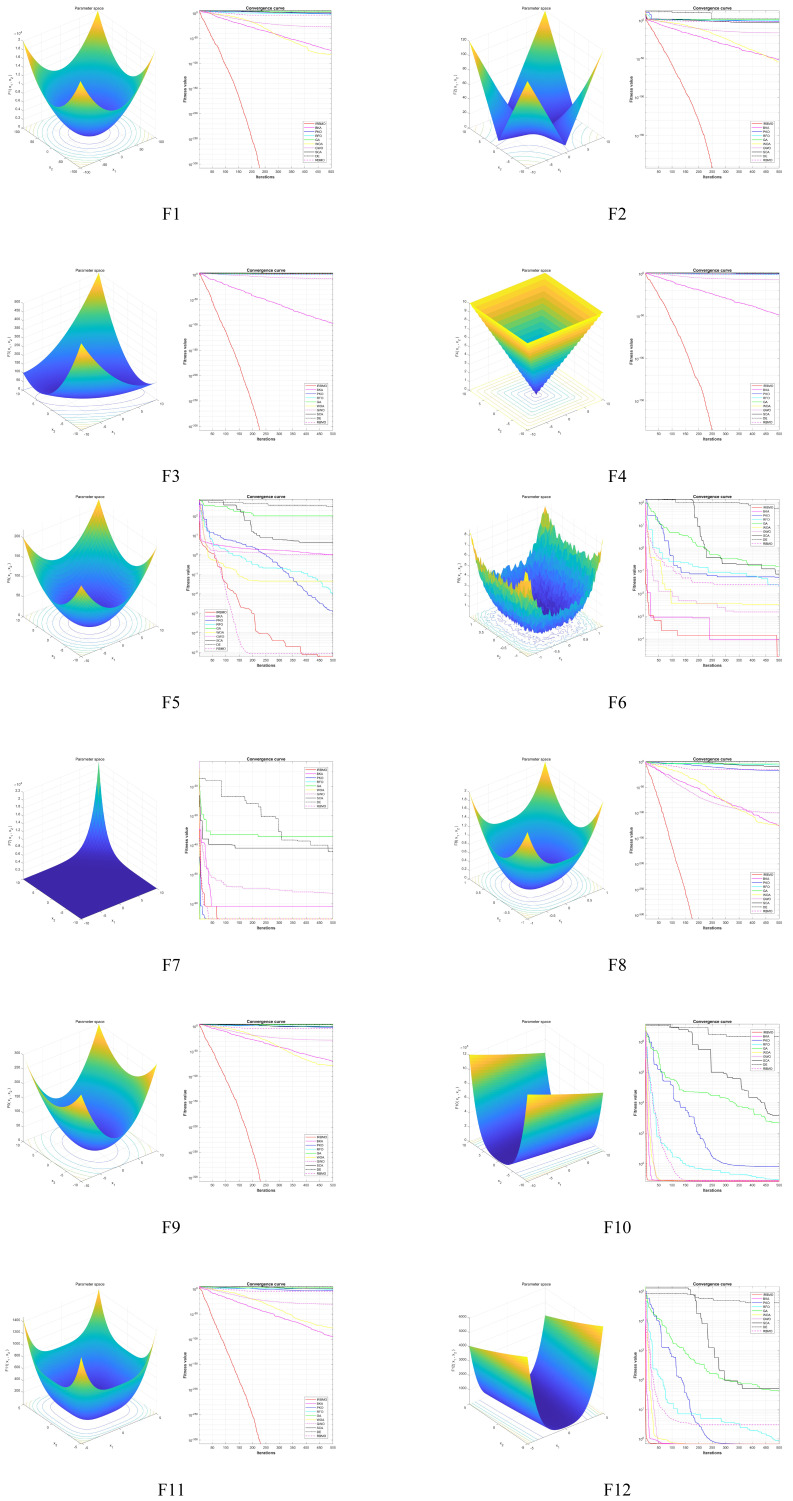

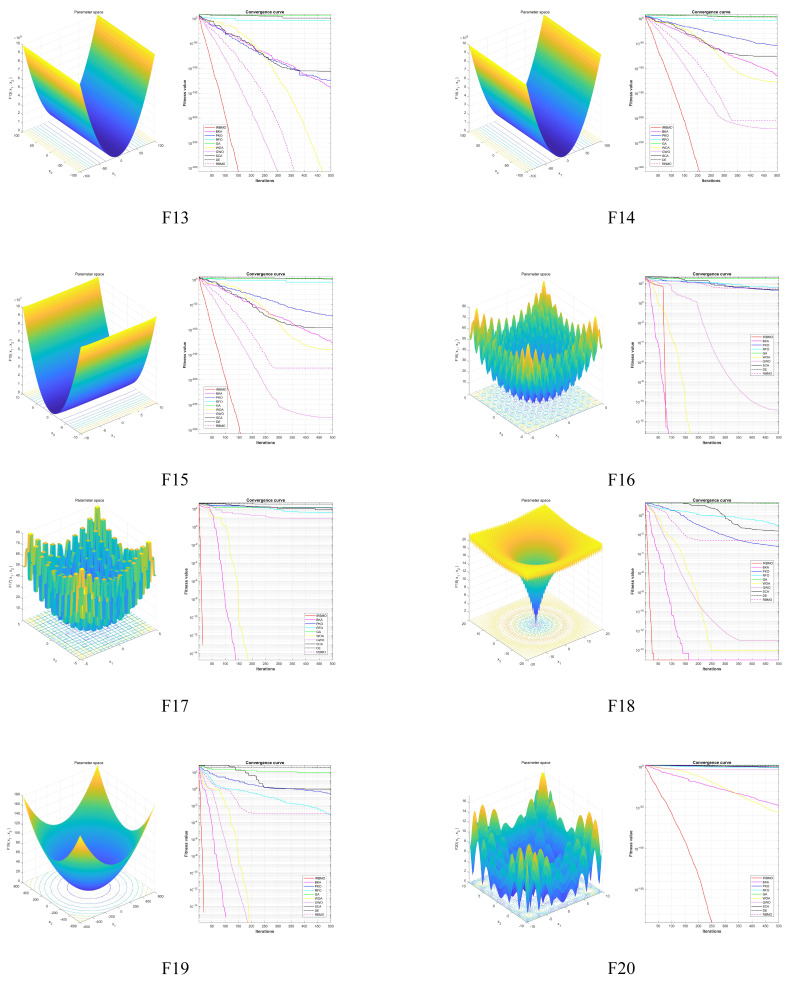

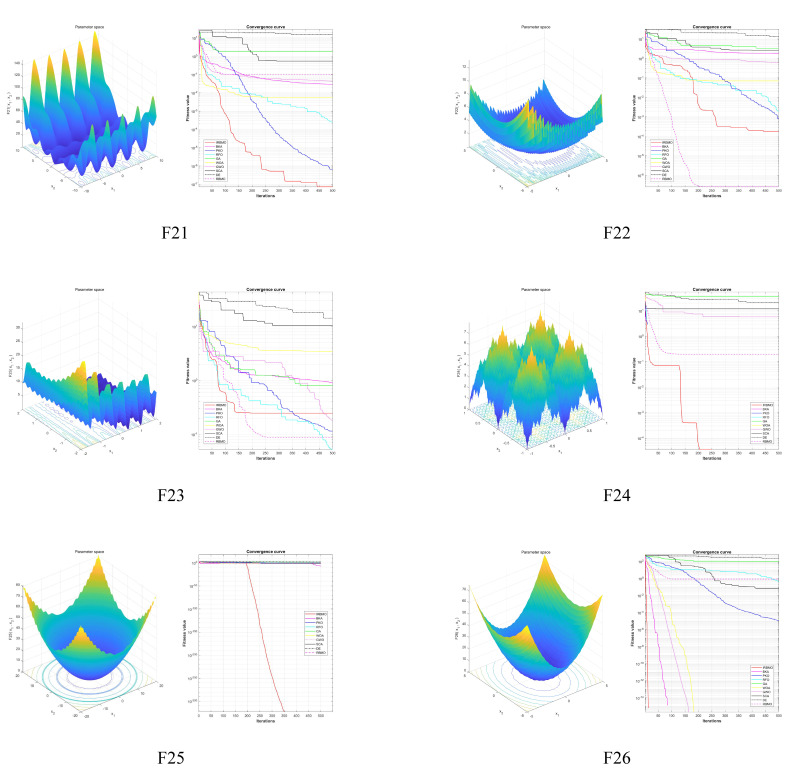
Convergence curves on each benchmark function.

The convergence characteristics of IRBMO, as illustrated in Figure 6, further support its superior performance. From the convergence plots, it is clear that IRBMO achieves a quicker convergence rate and more stable convergence behavior compared to competing approaches. Specifically, IRBMO shows steeper initial slopes on most test functions, indicating more efficient exploration of the problem domain during the initial phases of optimization. The algorithm maintains consistent progress toward optimal solutions with fewer oscillations and smoother convergence trajectories, particularly evident on complex multimodal functions such as F8, F9, F11, and F13. The convergence analysis reveals that IRBMO achieves superior final convergence values on the majority of test functions, with the algorithm consistently reaching lower fitness values compared to competing algorithms. This enhanced convergence behavior can be attributed to the synergistic interaction between the three core strategies and the effective balance between exploration and exploitation achieved through the multi-population cooperative framework. The elite guidance mechanism ensures that the algorithm maintains focus on promising regions, while adaptive differential evolution provides robust local search capabilities, and quasi-opposition learning prevents premature convergence by exploring alternative solution regions.

**Figure 7:**
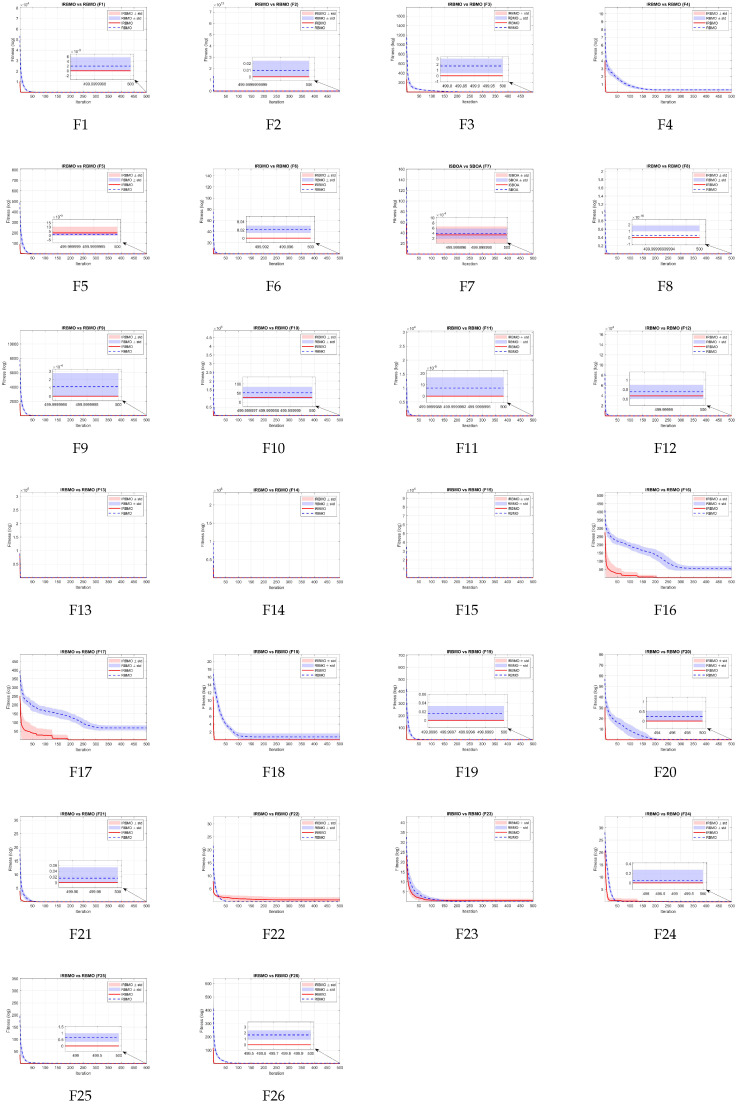
IRBMO vs. RBMO convergence curves on 26 benchmarks (mean ± std).

In addition, to clearly present IRBMO’s convergence speed and stability, Figure 7 reports the convergence curves of IRBMO and RBMO on 26 benchmark functions. IRBMO descends faster in the early iterations and attains lower final fitness on most tasks; its shaded bands (mean±std over 20 independent runs) are noticeably narrower, indicating smaller fluctuations and more stable convergence. The vertical axis uses a logarithmic scale to highlight differences at low values, and the horizontal axis denotes iterations.

In summary, the comprehensive experimental evaluation provides strong evidence of IRBMO’s superior optimization performance, statistical significance, and algorithmic robustness across diverse benchmark functions. These findings provide strong validation for the proposed improvement strategies. They also serve to demonstrate the algorithm’s potential when applied to complex real-world optimization problems.

### 4.2. Practical Engineering Applications

For practical validation, IRBMO is tested on four well-established engineering design problems [45,46,47,48]. These problems serve as benchmark test cases, and the performance of IRBMO is compared against that of several mainstream metaheuristic algorithms. The selected engineering problems include weight minimization of a speed reducer, step-cone pulley problem, gear train design problem, and rolling element bearing problem. To ensure a fair comparison, all algorithms were evaluated under the same experimental conditions. The population size was universally set to 30, and the maximum number of iterations was 500. Furthermore, to guarantee the statistical reliability of the findings, each experiment was conducted 20 times independently.

#### 4.2.1. Weight Minimization of a Speed Reducer

The weight minimization problem of a speed reducer aims to minimize the total weight while ensuring structural integrity and meeting all design constraints. This optimization problem seeks to determine seven design variables. The first three relate to the gear assembly: face width ($x_{1}$), module of teeth ($x_{2}$), and the number of teeth on the pinion ($x_{3}$). The remaining four define the shafts: the length of the first shaft ($x_{4}$) and second shaft ($x_{5}$) between bearings, and the diameters of the first shaft ($x_{6}$) and second shaft ($x_{7}$). The problem is subject to eleven nonlinear inequality constraints including bending stress, surface stress, transverse deflections, and geometric constraints. Given the complex multi-constraint nature and the practical engineering significance of this problem, it serves as an excellent benchmark for evaluating the performance of metaheuristic optimization algorithms.

Minimize

(30) $$f\left(x\right)=0.7854x_{1}x_{2}^{2}\left(3.3333x_{3}^{2}+14.9334x_{3}-43.0934\right)-1.508x_{1}\left(x_{6}^{2}+x_{7}^{2}\right)+7.477\left(x_{6}^{3}+x_{7}^{3}\right)+0.7854\left(x_{4}x_{6}^{2}+x_{5}x_{7}^{2}\right)$$

Subject to

$$\begin{array}{cc} g_{1}\left(x\right) & =-x_{1}x_{2}^{2}x_{3}+27\leq 0\\ g_{2}\left(x\right) & =-x_{1}x_{2}^{2}x_{3}^{2}+397.5\leq 0\\ g_{3}\left(x\right) & =-x_{2}x_{6}^{4}x_{3}x_{4}^{-3}+1.93\leq 0\\ g_{4}\left(x\right) & =-x_{2}x_{7}^{4}x_{3}/x_{5}^{3}+1.93\leq 0\\ g_{5}\left(x\right) & =10x_{6}^{-3}\sqrt{16.91\times 10^{6}+{\left(745x_{4}/\left(x_{2}x_{3}\right)\right)}^{2}}-1100\leq 0\\ g_{6}\left(x\right) & =10x_{7}^{-3}\sqrt{157.5\times 10^{6}+{\left(745x_{5}/\left(x_{2}x_{3}\right)\right)}^{2}}-850\leq 0\\ g_{7}\left(x\right) & =x_{2}x_{3}-40\leq 0\\ g_{8}\left(x\right) & =-x_{1}/x_{2}+5\leq 0\\ g_{9}\left(x\right) & =x_{1}/x_{2}-12\leq 0\\ g_{10}\left(x\right) & =1.5x_{6}-x_{4}+1.9\leq 0\\ g_{11}\left(x\right) & =1.1x_{7}-x_{5}+1.9\leq 0 \end{array}$$

With bounds

$$\begin{array}{c} 2.6\leq x_{1}\leq 3.6,\;0.7\leq x_{2}\leq 0.8,\;17\leq x_{3}\leq 28\\ 7.3\leq x_{4}\leq 8.3,\;7.3\leq x_{5}\leq 8.3,\;2.9\leq x_{6}\leq 3.9\\ 5.0\leq x_{7}\leq 5.5 \end{array}$$

#### 4.2.2. Step-Cone Pulley Problem

**Figure 8:**
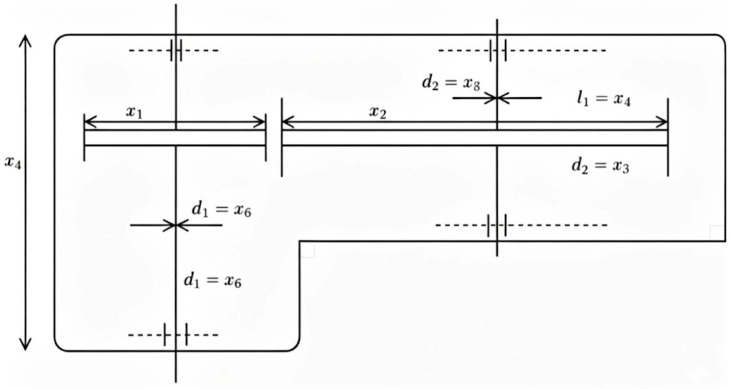
Schematic diagram of weight minimization of a speed reducer problem.

**Figure 9:**
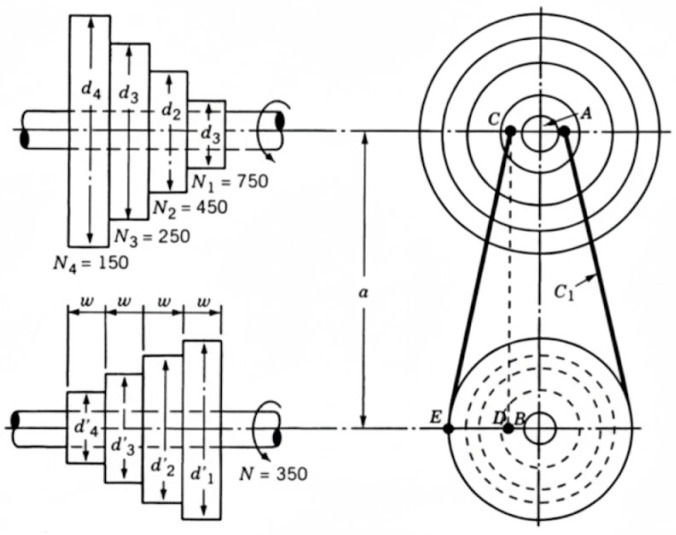
Schematic diagram of step-cone pulley problem.

This engineering task focuses on the step-cone pulley design. The primary objective is to reduce the system’s total weight, subject to constraints related to adequate power transmission and overall performance. A schematic diagram of the weight minimization of a speed reducer problem is shown in Figure 8. This optimization problem involves determining five design variables: diameters of the four steps (d1, d2, d3, d4) and the width of the pulley (w) (Figure 9). The problem is subject to eight nonlinear inequality constraints including power transmission requirements, geometric constraints, and manufacturing limitations. The complexity of this problem lies in the nonlinear relationships between pulley dimensions and power transmission capabilities, making it a challenging benchmark for optimization algorithms.

Minimize

(31) $$f\left(x\right)=\rho w\frac{\pi }{4}\left[d_{1}^{2}\left(1+{\left(\frac{N_{1}}{N}\right)}^{2}\right)+d_{2}^{2}\left(1+{\left(\frac{N_{2}}{N}\right)}^{2}\right)+d_{3}^{2}\left(1+{\left(\frac{N_{3}}{N}\right)}^{2}\right)+d_{4}^{2}\left(1+{\left(\frac{N_{4}}{N}\right)}^{2}\right)\right]$$

where ρ=7200 kg/m^3^, N=350 rpm, $N_{1}=750$ rpm, $N_{2}=450$ rpm, $N_{3}=250$ rpm, $N_{4}=150$ rpm.

Subject to

$$\begin{array}{cc} g_{1}\left(x\right) & =-R_{1}+2\leq 0\\ g_{2}\left(x\right) & =-R_{2}+2\leq 0\\ g_{3}\left(x\right) & =-R_{3}+2\leq 0\\ g_{4}\left(x\right) & =-R_{4}+2\leq 0\\ g_{5}\left(x\right) & =-P_{1}+\left(0.75\times 745.6998\right)\leq 0\\ g_{6}\left(x\right) & =-P_{2}+\left(0.75\times 745.6998\right)\leq 0\\ g_{7}\left(x\right) & =-P_{3}+\left(0.75\times 745.6998\right)\leq 0\\ g_{8}\left(x\right) & =-P_{4}+\left(0.75\times 745.6998\right)\leq 0 \end{array}$$

where $R_{i}=\exp\left(\mu \left(\pi -2\arcsin\left(\left(N_{i}/N-1\right)d_{i}/\left(2a\right)\right)\right)\right)$ and $P_{i}=stw\left(1-\exp\left(-\mu \left(\pi -2\arcsin\left(\left(N_{i}/N-1\right)d_{i}/\left(2a\right)\right)\right)\right)\right)\pi d_{i}N_{i}/60$ with μ=0.35, $s=1.75\times 10^{6}$ Pa, $t=8\times 10^{-3}$ m, a=3 m.

With bounds

$$0.02\leq d_{1},d_{2},d_{3},d_{4}\leq 0.5,\;\;\;0.01\leq w\leq 0.1$$

#### 4.2.3. Gear Train Design Problem

**Figure 10:**
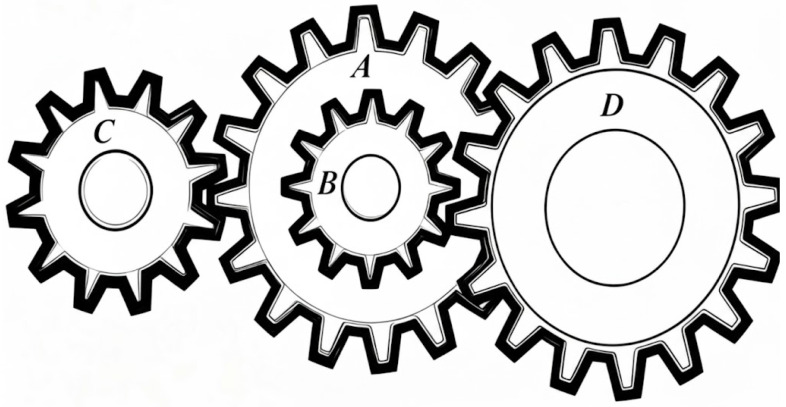
Schematic diagram of gear train design problem.

The gear train design problem requires determining the optimal number of teeth for four distinct gears ($x_{1},x_{2},x_{3}$, and $x_{4}$). The primary goal is to reduce the deviation between the resulting gear ratio and a target ratio of 1/6.931. This task is known for its simple objective function, yet it remains a fundamental challenge for mechanical design optimization. A diagram of the gear train is depicted in Figure 10. The discrete nature of gear teeth numbers and the need for precise gear ratio matching make this problem particularly suitable for testing the exploration and exploitation capabilities of optimization algorithms.

Minimize

(32) $$f\left(x\right)={\left(\frac{1}{6.931}-\frac{x_{1}x_{2}}{x_{3}x_{4}}\right)}^{2}$$

With bounds

$$\begin{array}{c} 12\leq x_{1},x_{2},x_{3},x_{4}\leq 60 \end{array}$$

#### 4.2.4. Rolling Element Bearing Problem

**Figure 11:**
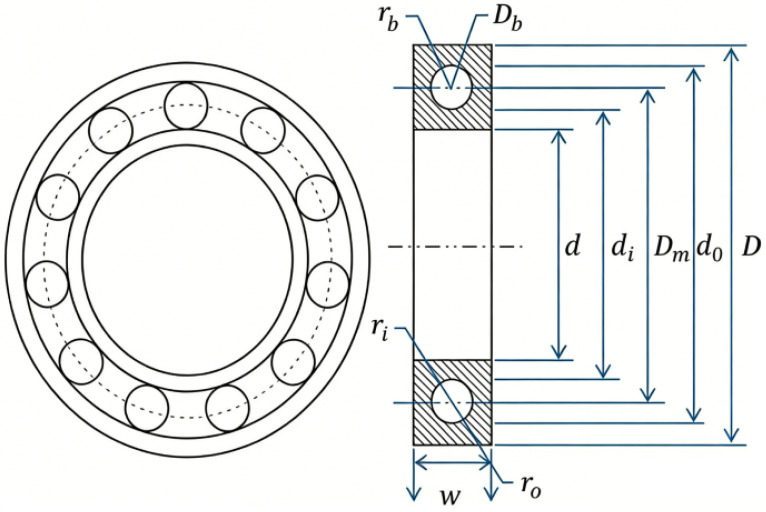
Schematic diagram of rolling element bearing.

This engineering problem focuses on designing a rolling element bearing. The primary objective is to minimize its dynamic capacity while adhering to various geometric and performance constraints. Figure 11 provides a schematic of this bearing. This optimization problem requires finding the optimal values for ten design variables. These are grouped as follows:

1. Main Dimensions: Mean diameter ($D_{m}$), ball diameter ($D_{b}$), and number of balls (*Z*).
2. Raceway Curvature: Inner raceway groove curvature radius coefficient ($f_{i}$) and outer raceway groove curvature radius coefficient ($f_{o}$).
3. Clearance and Load: Minimum diameter clearance ($KD_{min}$), maximum diameter clearance ($KD_{max}$), and the load distribution parameter (ϵ).
4. Other Parameters: Parameter (*e*) and parameter (χ). The problem is subject to eight nonlinear inequality constraints including geometric constraints, load capacity requirements, and manufacturing limitations. The complexity of this problem lies in the nonlinear relationships between bearing dimensions and dynamic capacity, making it a challenging benchmark for optimization algorithms in mechanical design applications.

Minimize

(33) $$f\left(x\right)=\left\{\begin{array}{cc} f_{c}\cdot Z^{2/3}\cdot D_{b}^{1.8} & \mathrm{if}\;D_{b}\leq 25.4\\ 3.647\cdot f_{c}\cdot Z^{2/3}\cdot D_{b}^{1.4} & \mathrm{if}\;D_{b}>25.4 \end{array}\right.$$

where $f_{c}=37.91{\left(1+{\left(1.04{\left(\left(1-\gamma \right)/\left(1+\gamma \right)\right)}^{1.72}{\left(f_{i}\left(2f_{o}-1\right)/\left(f_{o}\left(2f_{i}-1\right)\right)\right)}^{0.41}\right)}^{10/3}\right)}^{-0.3}$$\left(\gamma^{0.3}{\left(1-\gamma \right)}^{1.39}/{\left(1+\gamma \right)}^{1/3}\right){\left(2f_{i}/\left(2f_{i}-1\right)\right)}^{0.41}$ and $\gamma =D_{b}/D_{m}$.

Subject to

$$\begin{array}{cc} g_{1}\left(x\right) & =Z-1-\phi_{o}/\left(2\arcsin\left(D_{b}/D_{m}\right)\right)\leq 0\\ g_{2}\left(x\right) & =KD_{min}\left(D-d\right)-2D_{b}\leq 0\\ g_{3}\left(x\right) & =2D_{b}-KD_{max}\left(D-d\right)\leq 0\\ g_{4}\left(x\right) & =\chi B_{w}-D_{b}\leq 0\\ g_{5}\left(x\right) & =0.5\left(D+d\right)-D_{m}\leq 0\\ g_{6}\left(x\right) & =D_{m}-\left(0.5+e\right)\left(D+d\right)\leq 0\\ g_{7}\left(x\right) & =\epsilon D_{b}-0.5\left(D-D_{m}-D_{b}\right)\leq 0\\ g_{8}\left(x\right) & =0.515-f_{i}\leq 0\\ g_{9}\left(x\right) & =0.515-f_{o}\leq 0 \end{array}$$

where D=160 mm, d=90 mm, $B_{w}=30$ mm, $T=D-d-2D_{b}$, and $\phi_{o}=2\pi -2\arccos\left({\left(\left(D-d\right)0.5-0.75T\right)}^{2}+{\left(0.5D-0.25T-D_{b}\right)}^{2}-{\left(0.5d+0.25T\right)}^{2}\right)/\left(2\left(0.5\left(D-d\right)-0.75T\right)\left(0.5D-0.25T-D_{b}\right)\right)$.

With bounds

$$\begin{array}{c} 125\leq D_{m}\leq 150,\;10.5\leq D_{b}\leq 15.5,\;4\leq Z\leq 50\\ 0.515\leq f_{i},f_{o}\leq 0.6,\;0.4\leq KD_{min}\leq 0.5\\ 0.6\leq KD_{max}\leq 0.7,\;0.3\leq \epsilon \leq 0.4\\ 0.4\leq e\leq 0.5,\;0.6\leq \chi \leq 0.7 \end{array}$$

**Figure 12:**
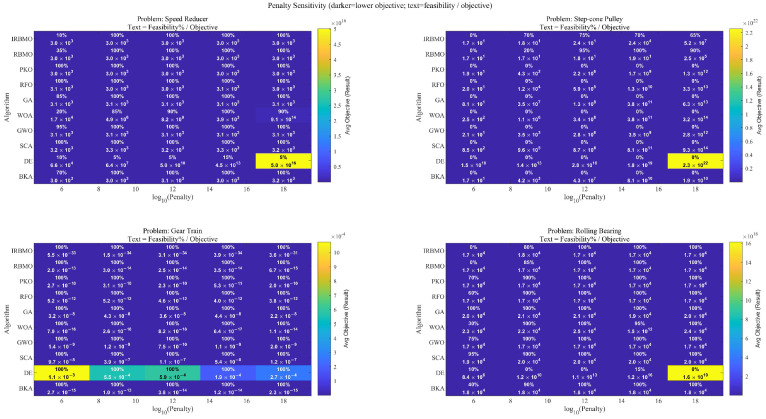
Penalty sensitivity for four engineering problems.

A penalty function approach is employed to manage the constraints associated with these engineering problems. This technique integrates a penalty for any constraint violations directly into the objective function, which is modified as follows:

(34) $$J_{aug}\left(x\right)=f\left(x\right)+\sum_{i=1}^{n}k_{i}\cdot b_{i}$$

where the penalty coefficient is represented by $k_{i}$. We performed a penalty-sensitivity analysis on the four engineering problems in this study (speed reducer, step-cone pulley, gear train, rolling bearing) and all algorithms, testing penalty values $10^{6}$, $10^{9}$, $10^{12}$, $10^{15}$, and $10^{18}$. The heatmap (color: average objective/result; darker is better; text: feasibility % /objective) shows that penalties $\geq 10^{12}$ achieve 100% feasibility with stable objectives. Among them, $10^{15}$ is the most robust choice across all problems and algorithms, simultaneously ensuring full feasibility and low objectives. Therefore, we adopt $k_{i}=10^{15}$ for all experiments. Selection rule: choose the smallest penalty that yields 100% feasibility and the lowest objective. The aggregated results are shown in Figure 12, while $b_{i}$ is defined as

(35) $$b_{i}=\left\{\begin{array}{cc} 0 & \mathrm{if}\;g_{i}\left(x\right)\leq 0\\ g_{i}{\left(x\right)}^{2} & \mathrm{if}\;g_{i}\left(x\right)>0 \end{array}\right.,\;i=1,2,\ldots ,n$$

As shown in Table 9, Table 10, Table 11 and Table 12, the superior performance of IRBMO is conclusively demonstrated across four benchmark engineering optimization problems: the weight minimization of a speed reducer, the step-cone pulley problem, the gear train design problem, and the rolling element bearing problem. Experimental results show that IRBMO consistently achieves the best objective function value in all four cases, significantly outperforming the other comparative algorithms.

This decisive advantage is rooted in IRBMO’s synergistic combination of three core strategies: enhanced RBMO search guided by elite solutions, adaptive differential evolution, and quasi-opposition-based learning. These mechanisms operate within a multi-population cooperative framework to ensure robust global exploration and intensified local exploitation. This cooperative and multifaceted approach enables IRBMO to effectively navigate the complex constraints and inherent multimodalities of real-world engineering design spaces, consistently achieving superior results compared to established optimization techniques.

## 5. Conclusions

The RBMO’s known deficiencies—premature convergence and an imbalanced exploration–exploitation mechanism—are addressed by the proposed IRBMO. This novel metaheuristic utilizes a multi-population cooperative framework that integrates three core strategies: enhanced RBMO search with elite guidance, an adaptive differential evolution operator, and quasi-opposition-based learning. Rigorous evaluation across 26 classical benchmark functions proved IRBMO’s substantial superiority over the original RBMO and other state-of-the-art algorithms in terms of solution accuracy and stability. Furthermore, IRBMO demonstrated exceptional robustness on four challenging real-world engineering design problems (including the weight minimization of a speed reducer, step-cone pulley, gear train, and rolling element bearing), consistently achieving globally optimal or highly competitive solutions.

For future work, the robust architecture of IRBMO can be extended to solve large-scale optimization problems (LSOPs) and multi-objective optimization problems (MOOPs), and its application can be explored in complex industrial domains like machine learning hyperparameter optimization.

## Figures and Tables

**Table 1:** The test functions.

Function Name	Formula	Range	$f_{\min}$
F1	$f_{1}\left(x\right)=\sum_{i=1}^{dim}x_{i}^{2}$	[−100,100]	0
F2	$f_{2}\left(x\right)=\sum_{i=1}^{dim}|x_{i}|+\prod_{i=1}^{dim}\left|x_{i}\right|$	[−10,10]	0
F3	$f_{3}\left(x\right)=\sum_{i=1}^{dim}{\left(\sum_{j=1}^{i}x_{j}\right)}^{2}$	[−10,10]	0
F4	$f_{4}\left(x\right)=\max_{i}\left\{\right|x_{i}\left|\right\}$	[−10,10]	0
F5	$f_{5}\left(x\right)=\sum_{i=1}^{dim}{\left|x_{i}+0.5\right|}^{2}$	[−10,10]	0
F6	$f_{6}\left(x\right)=\sum_{i=1}^{dim}ix_{i}^{4}+rand$	[−1.28,1.28]	0
F7	$f_{7}\left(x\right)=\exp\left(0.5\sum_{i=1}^{dim}x_{i}\right)$	[−10,10]	0
F8	$f_{8}\left(x\right)=\sum_{i=1}^{dim}{\left|x_{i}\right|}^{i+1}$	[−1,1]	0
F9	$f_{9}\left(x\right)=\sum_{i=1}^{dim}ix_{i}^{2}$	[−10,10]	0
F10	$f_{10}\left(x\right)=\sum_{i=1}^{dim-1}\left[100{\left(x_{i+1}-x_{i}^{2}\right)}^{2}+{\left(x_{i}-1\right)}^{2}\right]$	[−10,10]	0
F11	$f_{11}\left(x\right)=\sum_{i=1}^{dim}x_{i}^{2}+\left(\frac{D}{2}\sum_{i=1}^{dim}x_{i}^{2}\right)+\left(\frac{D}{2}\sum_{i=1}^{dim}x_{i}^{4}\right)$	[−5.12,5.12]	0
F12	$f_{12}\left(x\right)={\left(x_{1}-1\right)}^{2}+\sum_{i=2}^{dim}i{\left(2x_{i}^{2}-x_{i-1}\right)}^{2}$	[−5,5]	0
F13	$f_{13}\left(x\right)=\sum_{i=1}^{dim}{\left(10^{6}\right)}^{\frac{i-1}{dim-1}}x_{i}^{2}$	[−100,100]	0
F14	$f_{14}\left(x\right)=x_{1}^{2}+10^{6}\sum_{i=2}^{dim}x_{i}^{2}$	[−100,100]	0
F15	$f_{15}\left(x\right)=10^{6}x_{1}^{2}+\sum_{i=2}^{dim}x_{i}^{6}$	[−10,10]	0
F16	$f_{16}\left(x\right)=\sum_{i=1}^{dim}\left[x_{i}^{2}-10\cos\left(2\pi x_{i}\right)\right]+10dim$	[−5.12,5.12]	0
F17	$f_{17}\left(x\right)=\sum_{i=1}^{dim}y_{i}$, where $y_{i}=\left\{\begin{array}{cc} x_{i}^{2}-10\cos\left(2\pi x_{i}\right)+10 & |x_{i}|<0.5\\ {\left(\frac{round(2x_{i})}{2}\right)}^{2}-10\cos\left(2\pi \frac{round(2x_{i})}{2}\right)+10 & otherwise \end{array}\right.$	[−5.12,5.12]	0
F18	$f_{18}\left(x\right)=-20\exp\left(-0.2\sqrt{\frac{1}{dim}\sum_{i=1}^{dim}x_{i}^{2}}\right)-\exp\left(\frac{1}{dim}\sum_{i=1}^{dim}\cos\left(2\pi x_{i}\right)\right)+20+e$	[−20,20]	0
F19	$f_{19}\left(x\right)=\sum_{i=1}^{dim}\frac{x_{i}^{2}}{4000}-\prod_{i=1}^{dim}\cos\left(\frac{x_{i}}{\sqrt{i}}\right)+1$	[−600,600]	0
F20	$f_{20}\left(x\right)=\sum_{i=1}^{dim}\left|x_{i}\sin\left(x_{i}\right)+0.1x_{i}\right|$	[−10,10]	0
F21	$f_{21}\left(x\right)=\frac{\pi }{dim}\left[10\sin^{2}\left(\pi \left(1+\frac{x_{1}+1}{4}\right)\right)+\sum_{i=1}^{dim-1}\left({\left(\frac{x_{i}+1}{4}\right)}^{2}\left(1+10\sin^{2}\left(\pi \left(1+\frac{x_{i+1}+1}{4}\right)\right)\right)\right)+{\left(\frac{x_{dim}+1}{4}\right)}^{2}\right]+\sum_{i=1}^{dim}u\left(x_{i},10,100,4\right)$	[−10,10]	0
F22	$f_{22}\left(x\right)=0.1\left[\sin^{2}\left(3\pi x_{1}\right)+\sum_{i=1}^{dim-1}{\left(x_{i}-1\right)}^{2}\left(1+\sin^{2}\left(3\pi x_{i+1}\right)\right)+{\left(x_{dim}-1\right)}^{2}\left(1+\sin^{2}\left(2\pi x_{dim}\right)\right)\right]+\sum_{i=1}^{dim}u\left(x_{i},5,100,4\right)$	[−5,5]	0
F23	$f_{23}\left(x\right)=\sum_{i=1}^{dim-1}{\left(x_{i}-1\right)}^{2}\left(1+\sin^{2}\left(3\pi x_{i+1}\right)\right)+\sin^{2}\left(3\pi x_{1}\right)+{\left(x_{dim}-1\right)}^{2}\left(1+\sin^{2}\left(2\pi x_{dim}\right)\right)$	[−2,2]	0
F24	$f_{24}\left(x\right)=\sum_{i=1}^{dim}\left[\sum_{k=0}^{kmax}a^{k}\cos\left(2\pi b^{k}\left(x_{i}+0.5\right)\right)\right]-dim\sum_{k=0}^{kmax}a^{k}\cos\left(2\pi b^{k}\cdot 0.5\right)$	[−1,1]	0
F25	$f_{25}\left(x\right)=1-\cos\left(2\pi \sqrt{\sum_{i=1}^{dim}x_{i}^{2}}\right)+0.1\sum_{i=1}^{dim}x_{i}^{2}$	[−20,20]	0
F26	$f_{26}\left(x\right)=\sum_{i=1}^{dim-1}\left[x_{i}^{2}+2x_{i+1}^{2}-0.3\cos\left(2\pi x_{i}\right)-0.4\cos\left(4\pi x_{i+1}\right)+0.7\right]$	[−5,5]	0

**Table 2:** Detailed parameter descriptions of comparative algorithms.

Algorithms	Parameter Settings	Descriptions
IRBMO	Adaptive parameters (Epsilon, CF, mutation rate, crossover rate)	Parameters are dynamically adjusted according to function characteristics and iteration progress.
BKA	p=0.9, $n=0.05\times \exp(-2{\left(t/T\right)}^{2})$	*p* is attack probability; *n* is dynamically decaying parameter.
PKO	BF = 8, PE_*max*_ = 0.5, PE_*min*_ = 0	BF is beating factor; PE decreases linearly during iterations.
RFO	L=100, β=0.3×(1−t/T)	*L* is fixed parameter; β is dynamically decreasing step size factor.
GA/SGA	$p_{c}=0.7$, $p_{m}=1/dim$	$p_{c}$ is crossover probability; $p_{m}$ is mutation probability.
WOA	a=2−t×(2/T), b=1	*a* decreases linearly from 2 to 0; *b* is logarithmic spiral constant.
GWO	a=2−t×(2/T)	*a* decreases linearly from 2 to 0, controlling exploration and exploitation.
SCA	a=2, $r_{1}=a-t\times \left(a/T\right)$	*a* is initial value; $r_{1}$ decreases linearly.
DE	F=0.5, CR=0.5	*F* is mutation factor; CR is crossover probability.
RBMO	ϵ=0.5, $CF={\left(1-t/T\right)}^{2t/T}$	ϵ controls random selection; CF is dynamic convergence factor.

**Table 3:** Average fitness (Avg) results: IRBMO versus competing algorithms.

Func.	IRBMO	RBMO	PKO	RFO	GA	WOA	GWO	SCA	DE	BKA
F1	$0.000\times 10^{0}$	$5.621\times 10^{-6}$	$2.876\times 10^{-5}$	$7.597\times 10^{-3}$	$5.239\times 10^{3}$	$2.739\times 10^{-86}$	$3.893\times 10^{-29}$	$4.669\times 10^{-2}$	$2.475\times 10^{4}$	$2.407\times 10^{-2}$
F2	$0.000\times 10^{0}$	$2.054\times 10^{-4}$	$7.551\times 10^{-3}$	$1.838\times 10^{-1}$	$3.125\times 10^{1}$	$1.940\times 10^{-50}$	$1.050\times 10^{-16}$	$1.760\times 10^{-2}$	$2.608\times 10^{5}$	$1.090\times 10^{0}$
F3	$0.000\times 10^{0}$	$1.139\times 10^{-4}$	$2.197\times 10^{1}$	$7.722\times 10^{-1}$	$1.859\times 10^{2}$	$1.258\times 10^{2}$	$1.012\times 10^{-7}$	$8.312\times 10^{1}$	$7.598\times 10^{2}$	$1.786\times 10^{1}$
F4	$0.000\times 10^{0}$	$2.809\times 10^{-5}$	$5.659\times 10^{-1}$	$5.383\times 10^{-2}$	$7.171\times 10^{0}$	$2.756\times 10^{0}$	$1.096\times 10^{-7}$	$3.689\times 10^{0}$	$8.370\times 10^{0}$	$7.207\times 10^{-1}$
F5	$1.469\times 10^{0}$	$1.898\times 10^{0}$	$7.628\times 10^{-4}$	$1.060\times 10^{-2}$	$8.524\times 10^{1}$	$8.383\times 10^{-2}$	$6.835\times 10^{-1}$	$4.996\times 10^{0}$	$3.401\times 10^{2}$	$2.451\times 10^{-3}$
F6	$6.068\times 10^{-4}$	$2.693\times 10^{-4}$	$4.907\times 10^{-2}$	$3.213\times 10^{-2}$	$2.899\times 10^{-1}$	$4.543\times 10^{-3}$	$1.758\times 10^{-3}$	$1.492\times 10^{-1}$	$4.150\times 10^{1}$	$4.049\times 10^{-2}$
F7	$4.097\times 10^{-41}$	$1.988\times 10^{-32}$	$7.175\times 10^{-66}$	$7.175\times 10^{-66}$	$2.942\times 10^{-37}$	$7.175\times 10^{-66}$	$1.850\times 10^{-58}$	$1.709\times 10^{-40}$	$2.889\times 10^{-41}$	$3.834\times 10^{-54}$
F8	$6.166\times 10^{-117}$	$6.657\times 10^{-112}$	$8.385\times 10^{-10}$	$3.602\times 10^{-7}$	$3.109\times 10^{-5}$	$4.089\times 10^{-108}$	$1.060\times 10^{-97}$	$3.951\times 10^{-5}$	$2.703\times 10^{-1}$	$1.503\times 10^{-16}$
F9	$7.692\times 10^{-94}$	$1.451\times 10^{-77}$	$6.693\times 10^{-3}$	$2.183\times 10^{-1}$	$8.148\times 10^{2}$	$1.302\times 10^{-75}$	$2.313\times 10^{-28}$	$2.602\times 10^{0}$	$4.170\times 10^{3}$	$5.472\times 10^{-2}$
F10	$2.793\times 10^{1}$	$2.779\times 10^{1}$	$3.357\times 10^{1}$	$2.819\times 10^{1}$	$2.923\times 10^{3}$	$2.790\times 10^{1}$	$2.696\times 10^{1}$	$1.079\times 10^{3}$	$1.207\times 10^{6}$	$5.297\times 10^{1}$
F11	$3.216\times 10^{-81}$	$6.104\times 10^{-79}$	$2.692\times 10^{-2}$	$1.562\times 10^{-1}$	$1.184\times 10^{2}$	$4.962\times 10^{-75}$	$8.784\times 10^{-29}$	$8.712\times 10^{0}$	$1.373\times 10^{4}$	$2.037\times 10^{-2}$
F12	$6.973\times 10^{-1}$	$7.134\times 10^{-1}$	$7.568\times 10^{-1}$	$8.626\times 10^{-1}$	$9.968\times 10^{1}$	$6.671\times 10^{-1}$	$6.667\times 10^{-1}$	$4.219\times 10^{1}$	$4.085\times 10^{4}$	$3.189\times 10^{0}$
F13	$7.366\times 10^{-130}$	$6.674\times 10^{-129}$	$1.835\times 10^{-80}$	$2.845\times 10^{-5}$	$4.267\times 10^{4}$	$0.000\times 10^{0}$	$0.000\times 10^{0}$	$1.380\times 10^{-94}$	$2.380\times 10^{3}$	$1.907\times 10^{-25}$
F14	$2.567\times 10^{-108}$	$2.736\times 10^{-107}$	$1.332\times 10^{-46}$	$4.045\times 10^{-3}$	$3.243\times 10^{4}$	$1.524\times 10^{-101}$	$9.967\times 10^{-208}$	$1.723\times 10^{-66}$	$2.248\times 10^{3}$	$1.388\times 10^{-20}$
F15	$1.043\times 10^{-121}$	$5.494\times 10^{-123}$	$2.414\times 10^{-66}$	$1.704\times 10^{-4}$	$5.229\times 10^{2}$	$8.836\times 10^{-130}$	$3.992\times 10^{-262}$	$1.712\times 10^{-86}$	$3.461\times 10^{1}$	$1.650\times 10^{-27}$
F16	$0.000\times 10^{0}$	$0.000\times 10^{0}$	$3.055\times 10^{1}$	$2.620\times 10^{1}$	$2.530\times 10^{2}$	$8.527\times 10^{-15}$	$7.319\times 10^{0}$	$4.556\times 10^{1}$	$3.416\times 10^{2}$	$4.807\times 10^{1}$
F17	$0.000\times 10^{0}$	$0.000\times 10^{0}$	$4.829\times 10^{1}$	$3.323\times 10^{1}$	$1.566\times 10^{2}$	$0.000\times 10^{0}$	$8.402\times 10^{0}$	$7.327\times 10^{1}$	$3.138\times 10^{2}$	$4.846\times 10^{1}$
F18	$8.882\times 10^{-16}$	$8.882\times 10^{-16}$	$6.578\times 10^{-3}$	$9.205\times 10^{-2}$	$1.675\times 10^{1}$	$4.619\times 10^{-15}$	$9.379\times 10^{-14}$	$3.771\times 10^{-1}$	$1.651\times 10^{1}$	$4.428\times 10^{-1}$
F19	$0.000\times 10^{0}$	$0.000\times 10^{0}$	$7.410\times 10^{-2}$	$1.106\times 10^{-2}$	$7.466\times 10^{1}$	$0.000\times 10^{0}$	$3.726\times 10^{-3}$	$1.081\times 10^{0}$	$3.067\times 10^{2}$	$4.346\times 10^{-1}$
F20	$2.012\times 10^{-48}$	$1.693\times 10^{-40}$	$2.028\times 10^{-2}$	$1.180\times 10^{-1}$	$2.152\times 10^{1}$	$1.754\times 10^{-43}$	$5.267\times 10^{-4}$	$1.118\times 10^{0}$	$4.738\times 10^{1}$	$2.840\times 10^{-2}$
F21	$1.198\times 10^{-1}$	$6.094\times 10^{-2}$	$4.232\times 10^{-5}$	$1.766\times 10^{-4}$	$1.496\times 10^{0}$	$1.101\times 10^{-2}$	$3.793\times 10^{-2}$	$6.608\times 10^{-1}$	$1.218\times 10^{1}$	$5.707\times 10^{-2}$
F22	$1.820\times 10^{0}$	$1.650\times 10^{0}$	$4.319\times 10^{-3}$	$3.137\times 10^{-3}$	$2.944\times 10^{0}$	$1.782\times 10^{-1}$	$3.770\times 10^{-1}$	$2.325\times 10^{0}$	$1.298\times 10^{1}$	$6.696\times 10^{-4}$
F23	$2.209\times 10^{0}$	$2.513\times 10^{0}$	$1.724\times 10^{-1}$	$3.980\times 10^{-2}$	$4.616\times 10^{-1}$	$3.270\times 10^{-1}$	$6.565\times 10^{-1}$	$9.914\times 10^{0}$	$1.346\times 10^{1}$	$1.679\times 10^{-1}$
F24	$0.000\times 10^{0}$	$0.000\times 10^{0}$	$0.000\times 10^{0}$	$0.000\times 10^{0}$	$3.689\times 10^{1}$	$0.000\times 10^{0}$	$5.607\times 10^{0}$	$9.873\times 10^{0}$	$1.924\times 10^{1}$	$6.941\times 10^{-1}$
F25	$4.975\times 10^{-3}$	$4.977\times 10^{-3}$	$5.981\times 10^{-1}$	$3.570\times 10^{-1}$	$3.718\times 10^{1}$	$1.841\times 10^{-1}$	$2.637\times 10^{-1}$	$3.732\times 10^{-1}$	$1.428\times 10^{2}$	$9.612\times 10^{-1}$
F26	$0.000\times 10^{0}$	$0.000\times 10^{0}$	$1.530\times 10^{-3}$	$4.362\times 10^{-1}$	$9.334\times 10^{1}$	$0.000\times 10^{0}$	$0.000\times 10^{0}$	$2.118\times 10^{-1}$	$2.713\times 10^{2}$	$7.696\times 10^{-2}$

**Note:** The best results for each function are highlighted in **bold**.

**Table 4:** Optimal fitness (best) results: IRBMO versus competing algorithms.

Func.	IRBMO	RBMO	PKO	RFO	GA	WOA	GWO	SCA	DE	BKA
F1	$0.000\times 10^{0}$	$5.621\times 10^{-6}$	$2.876\times 10^{-5}$	$7.597\times 10^{-3}$	$5.239\times 10^{3}$	$2.739\times 10^{-86}$	$3.893\times 10^{-29}$	$4.669\times 10^{-2}$	$2.475\times 10^{4}$	$2.407\times 10^{-2}$
F2	$0.000\times 10^{0}$	$2.054\times 10^{-4}$	$7.551\times 10^{-3}$	$1.838\times 10^{-1}$	$3.125\times 10^{1}$	$1.940\times 10^{-50}$	$1.050\times 10^{-16}$	$1.760\times 10^{-2}$	$2.608\times 10^{5}$	$1.090\times 10^{0}$
F3	$0.000\times 10^{0}$	$1.139\times 10^{-4}$	$2.197\times 10^{1}$	$7.722\times 10^{-1}$	$1.859\times 10^{2}$	$1.258\times 10^{2}$	$1.012\times 10^{-7}$	$8.312\times 10^{1}$	$7.598\times 10^{2}$	$1.786\times 10^{1}$
F4	$0.000\times 10^{0}$	$2.809\times 10^{-5}$	$5.659\times 10^{-1}$	$5.383\times 10^{-2}$	$7.171\times 10^{0}$	$2.756\times 10^{0}$	$1.096\times 10^{-7}$	$3.689\times 10^{0}$	$8.370\times 10^{0}$	$7.207\times 10^{-1}$
F5	$1.469\times 10^{0}$	$1.898\times 10^{0}$	$7.628\times 10^{-4}$	$1.060\times 10^{-2}$	$8.524\times 10^{1}$	$8.383\times 10^{-2}$	$6.835\times 10^{-1}$	$4.996\times 10^{0}$	$3.401\times 10^{2}$	$2.451\times 10^{-3}$
F6	$6.068\times 10^{-4}$	$2.693\times 10^{-4}$	$4.907\times 10^{-2}$	$3.213\times 10^{-2}$	$2.899\times 10^{-1}$	$4.543\times 10^{-3}$	$1.758\times 10^{-3}$	$1.492\times 10^{-1}$	$4.150\times 10^{1}$	$4.049\times 10^{-2}$
F7	$4.097\times 10^{-41}$	$1.988\times 10^{-32}$	$7.175\times 10^{-66}$	$7.175\times 10^{-66}$	$2.942\times 10^{-37}$	$7.175\times 10^{-66}$	$1.850\times 10^{-58}$	$1.709\times 10^{-40}$	$2.889\times 10^{-41}$	$3.834\times 10^{-54}$
F8	$6.166\times 10^{-117}$	$6.657\times 10^{-112}$	$8.385\times 10^{-10}$	$3.602\times 10^{-7}$	$3.109\times 10^{-5}$	$4.089\times 10^{-108}$	$1.060\times 10^{-97}$	$3.951\times 10^{-5}$	$2.703\times 10^{-1}$	$1.503\times 10^{-16}$
F9	$7.692\times 10^{-94}$	$1.451\times 10^{-77}$	$6.693\times 10^{-3}$	$2.183\times 10^{-1}$	$8.148\times 10^{2}$	$1.302\times 10^{-75}$	$2.313\times 10^{-28}$	$2.602\times 10^{0}$	$4.170\times 10^{3}$	$5.472\times 10^{-2}$
F10	$2.793\times 10^{1}$	$2.779\times 10^{1}$	$3.357\times 10^{1}$	$2.819\times 10^{1}$	$2.923\times 10^{3}$	$2.790\times 10^{1}$	$2.696\times 10^{1}$	$1.079\times 10^{3}$	$1.207\times 10^{6}$	$5.297\times 10^{1}$
F11	$3.216\times 10^{-81}$	$6.104\times 10^{-79}$	$2.692\times 10^{-2}$	$1.562\times 10^{-1}$	$1.184\times 10^{2}$	$4.962\times 10^{-75}$	$8.784\times 10^{-29}$	$8.712\times 10^{0}$	$1.373\times 10^{4}$	$2.037\times 10^{-2}$
F12	$6.973\times 10^{-1}$	$7.134\times 10^{-1}$	$7.568\times 10^{-1}$	$8.626\times 10^{-1}$	$9.968\times 10^{1}$	$6.671\times 10^{-1}$	$6.667\times 10^{-1}$	$4.219\times 10^{1}$	$4.085\times 10^{4}$	$3.189\times 10^{0}$
F13	$7.366\times 10^{-130}$	$6.674\times 10^{-129}$	$1.835\times 10^{-80}$	$2.845\times 10^{-5}$	$4.267\times 10^{4}$	$0.000\times 10^{0}$	$0.000\times 10^{0}$	$1.380\times 10^{-94}$	$2.380\times 10^{3}$	$1.907\times 10^{-25}$
F14	$2.567\times 10^{-108}$	$2.736\times 10^{-107}$	$1.332\times 10^{-46}$	$4.045\times 10^{-3}$	$3.243\times 10^{4}$	$1.524\times 10^{-101}$	$9.967\times 10^{-208}$	$1.723\times 10^{-66}$	$2.248\times 10^{3}$	$1.388\times 10^{-20}$
F15	$1.043\times 10^{-121}$	$5.494\times 10^{-123}$	$2.414\times 10^{-66}$	$1.704\times 10^{-4}$	$5.229\times 10^{2}$	$8.836\times 10^{-130}$	$3.992\times 10^{-262}$	$1.712\times 10^{-86}$	$3.461\times 10^{1}$	$1.650\times 10^{-27}$
F16	$0.000\times 10^{0}$	$0.000\times 10^{0}$	$3.055\times 10^{1}$	$2.620\times 10^{1}$	$2.530\times 10^{2}$	$8.527\times 10^{-15}$	$7.319\times 10^{0}$	$4.556\times 10^{1}$	$3.416\times 10^{2}$	$4.807\times 10^{1}$
F17	$0.000\times 10^{0}$	$0.000\times 10^{0}$	$4.829\times 10^{1}$	$3.323\times 10^{1}$	$1.566\times 10^{2}$	$0.000\times 10^{0}$	$8.402\times 10^{0}$	$7.327\times 10^{1}$	$3.138\times 10^{2}$	$4.846\times 10^{1}$
F18	$8.882\times 10^{-16}$	$8.882\times 10^{-16}$	$6.578\times 10^{-3}$	$9.205\times 10^{-2}$	$1.675\times 10^{1}$	$4.619\times 10^{-15}$	$9.379\times 10^{-14}$	$3.771\times 10^{-1}$	$1.651\times 10^{1}$	$4.428\times 10^{-1}$
F19	$0.000\times 10^{0}$	$0.000\times 10^{0}$	$7.410\times 10^{-2}$	$1.106\times 10^{-2}$	$7.466\times 10^{1}$	$0.000\times 10^{0}$	$3.726\times 10^{-3}$	$1.081\times 10^{0}$	$3.067\times 10^{2}$	$4.346\times 10^{-1}$
F20	$2.012\times 10^{-48}$	$1.693\times 10^{-40}$	$2.028\times 10^{-2}$	$1.180\times 10^{-1}$	$2.152\times 10^{1}$	$1.754\times 10^{-43}$	$5.267\times 10^{-4}$	$1.118\times 10^{0}$	$4.738\times 10^{1}$	$2.840\times 10^{-2}$
F21	$1.198\times 10^{-1}$	$6.094\times 10^{-2}$	$4.232\times 10^{-5}$	$1.766\times 10^{-4}$	$1.496\times 10^{0}$	$1.101\times 10^{-2}$	$3.793\times 10^{-2}$	$6.608\times 10^{-1}$	$1.218\times 10^{1}$	$5.707\times 10^{-2}$
F22	$1.820\times 10^{0}$	$1.650\times 10^{0}$	$4.319\times 10^{-3}$	$3.137\times 10^{-3}$	$2.944\times 10^{0}$	$1.782\times 10^{-1}$	$3.770\times 10^{-1}$	$2.325\times 10^{0}$	$1.298\times 10^{1}$	$6.696\times 10^{-4}$
F23	$2.209\times 10^{0}$	$2.513\times 10^{0}$	$1.724\times 10^{-1}$	$3.980\times 10^{-2}$	$4.616\times 10^{-1}$	$3.270\times 10^{-1}$	$6.565\times 10^{-1}$	$9.914\times 10^{0}$	$1.346\times 10^{1}$	$1.679\times 10^{-1}$
F24	$0.000\times 10^{0}$	$0.000\times 10^{0}$	$0.000\times 10^{0}$	$0.000\times 10^{0}$	$3.689\times 10^{1}$	$0.000\times 10^{0}$	$5.607\times 10^{0}$	$9.873\times 10^{0}$	$1.924\times 10^{1}$	$6.941\times 10^{-1}$
F25	$4.975\times 10^{-3}$	$4.977\times 10^{-3}$	$5.981\times 10^{-1}$	$3.570\times 10^{-1}$	$3.718\times 10^{1}$	$1.841\times 10^{-1}$	$2.637\times 10^{-1}$	$3.732\times 10^{-1}$	$1.428\times 10^{2}$	$9.612\times 10^{-1}$
F26	$0.000\times 10^{0}$	$0.000\times 10^{0}$	$1.530\times 10^{-3}$	$4.362\times 10^{-1}$	$9.334\times 10^{1}$	$0.000\times 10^{0}$	$0.000\times 10^{0}$	$2.118\times 10^{-1}$	$2.713\times 10^{2}$	$7.696\times 10^{-2}$

**Note:** The best results for each function are highlighted in **bold**.

**Table 5:** Results of statistical significance tests.

Func.	RBMO	PKO	RFO	GA	WOA	GWO	SCA	DE	BKA
F1	$2.853\times 10^{-2}$	$6.796\times 10^{-8}$	$6.796\times 10^{-8}$	$6.796\times 10^{-8}$	$1.235\times 10^{-7}$	$6.796\times 10^{-8}$	$6.796\times 10^{-8}$	$6.796\times 10^{-8}$	$6.796\times 10^{-8}$
F2	$4.353\times 10^{-2}$	$6.796\times 10^{-8}$	$6.796\times 10^{-8}$	$6.796\times 10^{-8}$	$2.416\times 10^{-7}$	$6.796\times 10^{-8}$	$6.796\times 10^{-8}$	$6.796\times 10^{-8}$	$6.796\times 10^{-8}$
F3	$8.920\times 10^{-2}$	$6.796\times 10^{-8}$	$6.796\times 10^{-8}$	$6.796\times 10^{-8}$	$6.796\times 10^{-8}$	$6.796\times 10^{-8}$	$6.796\times 10^{-8}$	$6.796\times 10^{-8}$	$6.796\times 10^{-8}$
F4	$1.794\times 10^{-2}$	$6.796\times 10^{-8}$	$6.796\times 10^{-8}$	$6.796\times 10^{-8}$	$6.796\times 10^{-8}$	$6.796\times 10^{-8}$	$6.796\times 10^{-8}$	$6.796\times 10^{-8}$	$6.796\times 10^{-8}$
F5	$2.616\times 10^{-2}$	$6.796\times 10^{-8}$	$6.796\times 10^{-8}$	$6.796\times 10^{-8}$	$6.796\times 10^{-8}$	$3.293\times 10^{-5}$	$6.796\times 10^{-8}$	$6.796\times 10^{-8}$	$6.796\times 10^{-8}$
F6	$2.977\times 10^{-1}$	$6.796\times 10^{-8}$	$6.796\times 10^{-8}$	$6.796\times 10^{-8}$	$4.680\times 10^{-5}$	$6.674\times 10^{-6}$	$6.796\times 10^{-8}$	$6.796\times 10^{-8}$	$6.796\times 10^{-8}$
F7	$9.023\times 10^{-1}$	$9.029\times 10^{-8}$	$9.029\times 10^{-8}$	$4.654\times 10^{-7}$	$9.029\times 10^{-5}$	$1.993\times 10^{-2}$	$1.659\times 10^{-5}$	$7.709\times 10^{-5}$	$7.761\times 10^{-3}$
F8	$6.750\times 10^{-1}$	$6.796\times 10^{-8}$	$6.796\times 10^{-8}$	$6.796\times 10^{-8}$	$4.320\times 10^{-3}$	$6.796\times 10^{-8}$	$6.796\times 10^{-8}$	$6.796\times 10^{-8}$	$6.796\times 10^{-8}$
F9	$1.428\times 10^{-2}$	$6.796\times 10^{-8}$	$6.796\times 10^{-8}$	$6.796\times 10^{-8}$	$6.796\times 10^{-8}$	$6.796\times 10^{-8}$	$6.796\times 10^{-8}$	$6.796\times 10^{-8}$	$6.796\times 10^{-8}$
F10	$5.428\times 10^{-1}$	$3.750\times 10^{-4}$	$2.041\times 10^{-5}$	$6.796\times 10^{-8}$	$6.359\times 10^{-1}$	$2.343\times 10^{-3}$	$6.796\times 10^{-8}$	$6.796\times 10^{-8}$	$1.782\times 10^{-3}$
F11	$2.564\times 10^{-2}$	$6.796\times 10^{-8}$	$6.796\times 10^{-8}$	$6.796\times 10^{-8}$	$2.218\times 10^{-7}$	$6.796\times 10^{-8}$	$6.796\times 10^{-8}$	$6.796\times 10^{-8}$	$6.796\times 10^{-8}$
F12	$8.817\times 10^{-1}$	$1.250\times 10^{-5}$	$9.748\times 10^{-5}$	$6.796\times 10^{-8}$	$1.807\times 10^{-5}$	$4.680\times 10^{-5}$	$9.173\times 10^{-8}$	$6.796\times 10^{-8}$	$2.960\times 10^{-7}$
F13	$2.229\times 10^{-2}$	$3.416\times 10^{-7}$	$6.796\times 10^{-8}$	$6.796\times 10^{-8}$	$8.007\times 10^{-9}$	$8.007\times 10^{-9}$	$6.796\times 10^{-8}$	$6.796\times 10^{-8}$	$6.796\times 10^{-8}$
F14	$3.554\times 10^{-2}$	$6.796\times 10^{-8}$	$6.796\times 10^{-8}$	$6.796\times 10^{-8}$	$6.868\times 10^{-4}$	$6.796\times 10^{-8}$	$6.796\times 10^{-8}$	$6.796\times 10^{-8}$	$6.796\times 10^{-8}$
F15	$4.964\times 10^{-2}$	$6.796\times 10^{-8}$	$6.796\times 10^{-8}$	$6.796\times 10^{-8}$	$7.948\times 10^{-7}$	$6.796\times 10^{-8}$	$6.796\times 10^{-8}$	$6.796\times 10^{-8}$	$6.796\times 10^{-8}$
F16	$1.000\times 10^{0}$	$8.007\times 10^{-9}$	$8.007\times 10^{-9}$	$8.007\times 10^{-9}$	$8.036\times 10^{-2}$	$7.803\times 10^{-9}$	$8.007\times 10^{-9}$	$8.007\times 10^{-9}$	$8.007\times 10^{-9}$
F17	$1.000\times 10^{0}$	$8.007\times 10^{-9}$	$7.992\times 10^{-9}$	$8.007\times 10^{-9}$	$1.000\times 10^{0}$	$8.007\times 10^{-9}$	$8.007\times 10^{-9}$	$8.007\times 10^{-9}$	$8.007\times 10^{-9}$
F18	$1.000\times 10^{0}$	$8.007\times 10^{-9}$	$8.007\times 10^{-9}$	$8.007\times 10^{-9}$	$7.675\times 10^{-7}$	$7.732\times 10^{-9}$	$8.007\times 10^{-9}$	$8.007\times 10^{-9}$	$8.007\times 10^{-9}$
F19	$1.000\times 10^{0}$	$8.007\times 10^{-9}$	$8.007\times 10^{-9}$	$8.007\times 10^{-9}$	$1.000\times 10^{0}$	$1.980\times 10^{-2}$	$8.007\times 10^{-9}$	$8.007\times 10^{-9}$	$8.007\times 10^{-9}$
F20	$4.903\times 10^{-2}$	$6.796\times 10^{-8}$	$6.796\times 10^{-8}$	$6.796\times 10^{-8}$	$8.357\times 10^{-4}$	$6.796\times 10^{-8}$	$6.796\times 10^{-8}$	$6.796\times 10^{-8}$	$6.796\times 10^{-8}$
F21	$5.428\times 10^{-1}$	$6.796\times 10^{-8}$	$6.796\times 10^{-8}$	$1.657\times 10^{-7}$	$1.918\times 10^{-7}$	$4.320\times 10^{-3}$	$1.235\times 10^{-7}$	$6.796\times 10^{-8}$	$6.389\times 10^{-2}$
F22	$5.250\times 10^{-1}$	$6.796\times 10^{-8}$	$6.796\times 10^{-8}$	$1.190\times 10^{-4}$	$6.796\times 10^{-8}$	$7.898\times 10^{-8}$	$7.718\times 10^{-3}$	$6.796\times 10^{-8}$	$6.796\times 10^{-8}$
F23	$7.150\times 10^{-1}$	$2.960\times 10^{-7}$	$6.796\times 10^{-8}$	$1.190\times 10^{-4}$	$1.104\times 10^{-5}$	$7.718\times 10^{-3}$	$2.356\times 10^{-6}$	$7.948\times 10^{-7}$	$1.918\times 10^{-7}$
F24	$1.000\times 10^{0}$	$1.000\times 10^{0}$	$1.000\times 10^{0}$	$8.007\times 10^{-9}$	$1.000\times 10^{0}$	$8.007\times 10^{-9}$	$8.007\times 10^{-9}$	$8.007\times 10^{-9}$	$8.007\times 10^{-9}$
F25	$4.903\times 10^{-1}$	$6.796\times 10^{-8}$	$6.796\times 10^{-8}$	$6.796\times 10^{-8}$	$3.288\times 10^{-5}$	$6.796\times 10^{-8}$	$6.796\times 10^{-8}$	$6.796\times 10^{-8}$	$6.796\times 10^{-8}$
F26	$1.000\times 10^{0}$	$8.007\times 10^{-9}$	$8.007\times 10^{-9}$	$8.007\times 10^{-9}$	$1.000\times 10^{0}$	$1.000\times 10^{0}$	$8.007\times 10^{-9}$	$8.007\times 10^{-9}$	$8.007\times 10^{-9}$

**Note:** Significant results are highlighted in **bold**.

**Table 6:** Bonferroni-corrected *p*-values for all pairwise comparisons.

Func.	RBMO	PKO	RFO	GA	WOA	GWO	SCA	DE	BKA
F1	$6.680\times 10^{0}$	$1.591\times 10^{-5}$	$1.591\times 10^{-5}$	$1.591\times 10^{-5}$	$2.890\times 10^{-5}$	$1.591\times 10^{-5}$	$1.591\times 10^{-5}$	$1.591\times 10^{-5}$	$1.591\times 10^{-5}$
F2	$1.019\times 10^{1}$	$1.591\times 10^{-5}$	$1.591\times 10^{-5}$	$1.591\times 10^{-5}$	$5.653\times 10^{-5}$	$1.591\times 10^{-5}$	$1.591\times 10^{-5}$	$1.591\times 10^{-5}$	$1.591\times 10^{-5}$
F3	$2.089\times 10^{1}$	$1.591\times 10^{-5}$	$1.591\times 10^{-5}$	$1.591\times 10^{-5}$	$1.591\times 10^{-5}$	$1.591\times 10^{-5}$	$1.591\times 10^{-5}$	$1.591\times 10^{-5}$	$1.591\times 10^{-5}$
F4	$4.201\times 10^{0}$	$1.591\times 10^{-5}$	$1.591\times 10^{-5}$	$1.591\times 10^{-5}$	$1.591\times 10^{-5}$	$1.591\times 10^{-5}$	$1.591\times 10^{-5}$	$1.591\times 10^{-5}$	$1.591\times 10^{-5}$
F5	$6.121\times 10^{0}$	$1.591\times 10^{-5}$	$1.591\times 10^{-5}$	$1.591\times 10^{-5}$	$1.591\times 10^{-5}$	$7.706\times 10^{-3}$	$1.591\times 10^{-5}$	$1.591\times 10^{-5}$	$1.591\times 10^{-5}$
F6	$6.966\times 10^{1}$	$1.591\times 10^{-5}$	$1.591\times 10^{-5}$	$1.591\times 10^{-5}$	$1.095\times 10^{-2}$	$1.563\times 10^{-3}$	$1.591\times 10^{-5}$	$1.591\times 10^{-5}$	$1.591\times 10^{-5}$
F7	$2.111\times 10^{2}$	$2.114\times 10^{-5}$	$2.114\times 10^{-5}$	$1.089\times 10^{-4}$	$2.114\times 10^{-2}$	$4.664\times 10^{0}$	$3.888\times 10^{-3}$	$1.804\times 10^{-2}$	$1.816\times 10^{0}$
F8	$1.581\times 10^{2}$	$1.591\times 10^{-5}$	$1.591\times 10^{-5}$	$1.591\times 10^{-5}$	$1.011\times 10^{0}$	$1.591\times 10^{-5}$	$1.591\times 10^{-5}$	$1.591\times 10^{-5}$	$1.591\times 10^{-5}$
F9	$3.342\times 10^{0}$	$1.591\times 10^{-5}$	$1.591\times 10^{-5}$	$1.591\times 10^{-5}$	$1.591\times 10^{-5}$	$1.591\times 10^{-5}$	$1.591\times 10^{-5}$	$1.591\times 10^{-5}$	$1.591\times 10^{-5}$
F10	$1.270\times 10^{2}$	$8.775\times 10^{-2}$	$4.776\times 10^{-3}$	$1.591\times 10^{-5}$	$1.488\times 10^{2}$	$5.483\times 10^{-1}$	$1.591\times 10^{-5}$	$1.591\times 10^{-5}$	$4.170\times 10^{-1}$
F11	$6.000\times 10^{0}$	$1.591\times 10^{-5}$	$1.591\times 10^{-5}$	$1.591\times 10^{-5}$	$5.190\times 10^{-5}$	$1.591\times 10^{-5}$	$1.591\times 10^{-5}$	$1.591\times 10^{-5}$	$1.591\times 10^{-5}$
F12	$2.063\times 10^{2}$	$2.925\times 10^{-3}$	$2.281\times 10^{-2}$	$1.591\times 10^{-5}$	$4.228\times 10^{-3}$	$1.095\times 10^{-2}$	$2.146\times 10^{-5}$	$1.591\times 10^{-5}$	$6.926\times 10^{-5}$
F13	$5.216\times 10^{0}$	$8.000\times 10^{-5}$	$1.591\times 10^{-5}$	$1.591\times 10^{-5}$	$1.874\times 10^{-6}$	$1.874\times 10^{-6}$	$1.591\times 10^{-5}$	$1.591\times 10^{-5}$	$1.591\times 10^{-5}$
F14	$8.316\times 10^{0}$	$1.591\times 10^{-5}$	$1.591\times 10^{-5}$	$1.591\times 10^{-5}$	$1.608\times 10^{-1}$	$1.591\times 10^{-5}$	$1.591\times 10^{-5}$	$1.591\times 10^{-5}$	$1.591\times 10^{-5}$
F15	$1.163\times 10^{1}$	$1.591\times 10^{-5}$	$1.591\times 10^{-5}$	$1.591\times 10^{-5}$	$1.860\times 10^{-4}$	$1.591\times 10^{-5}$	$1.591\times 10^{-5}$	$1.591\times 10^{-5}$	$1.591\times 10^{-5}$
F16	$2.340\times 10^{2}$	$1.874\times 10^{-6}$	$1.874\times 10^{-6}$	$1.874\times 10^{-6}$	$1.880\times 10^{1}$	$1.826\times 10^{-6}$	$1.874\times 10^{-6}$	$1.874\times 10^{-6}$	$1.874\times 10^{-6}$
F17	$2.340\times 10^{2}$	$1.874\times 10^{-6}$	$1.871\times 10^{-6}$	$1.874\times 10^{-6}$	$2.340\times 10^{2}$	$1.874\times 10^{-6}$	$1.874\times 10^{-6}$	$1.874\times 10^{-6}$	$1.874\times 10^{-6}$
F18	$2.340\times 10^{2}$	$1.874\times 10^{-6}$	$1.874\times 10^{-6}$	$1.874\times 10^{-6}$	$1.798\times 10^{-4}$	$1.811\times 10^{-6}$	$1.874\times 10^{-6}$	$1.874\times 10^{-6}$	$1.874\times 10^{-6}$
F19	$2.340\times 10^{2}$	$1.874\times 10^{-6}$	$1.874\times 10^{-6}$	$1.874\times 10^{-6}$	$2.340\times 10^{2}$	$4.633\times 10^{0}$	$1.874\times 10^{-6}$	$1.874\times 10^{-6}$	$1.874\times 10^{-6}$
F20	$1.163\times 10^{1}$	$1.591\times 10^{-5}$	$1.591\times 10^{-5}$	$1.591\times 10^{-5}$	$1.956\times 10^{-1}$	$1.591\times 10^{-5}$	$1.591\times 10^{-5}$	$1.591\times 10^{-5}$	$1.591\times 10^{-5}$
F21	$1.270\times 10^{2}$	$1.591\times 10^{-5}$	$1.591\times 10^{-5}$	$3.877\times 10^{-5}$	$4.488\times 10^{-5}$	$1.011\times 10^{0}$	$2.890\times 10^{-5}$	$1.591\times 10^{-5}$	$1.496\times 10^{1}$
F22	$1.229\times 10^{2}$	$1.591\times 10^{-5}$	$1.591\times 10^{-5}$	$2.785\times 10^{-2}$	$1.591\times 10^{-5}$	$1.850\times 10^{-5}$	$1.806\times 10^{0}$	$1.591\times 10^{-5}$	$1.591\times 10^{-5}$
F23	$1.673\times 10^{2}$	$6.926\times 10^{-5}$	$1.591\times 10^{-5}$	$2.785\times 10^{-2}$	$2.583\times 10^{-3}$	$1.806\times 10^{0}$	$5.513\times 10^{-4}$	$1.860\times 10^{-4}$	$4.488\times 10^{-5}$
F24	$2.340\times 10^{2}$	$2.340\times 10^{2}$	$2.340\times 10^{2}$	$1.874\times 10^{-6}$	$2.340\times 10^{2}$	$1.874\times 10^{-6}$	$1.874\times 10^{-6}$	$1.874\times 10^{-6}$	$1.874\times 10^{-6}$
F25	$1.147\times 10^{2}$	$1.591\times 10^{-5}$	$1.591\times 10^{-5}$	$1.591\times 10^{-5}$	$7.694\times 10^{-3}$	$1.591\times 10^{-5}$	$1.591\times 10^{-5}$	$1.591\times 10^{-5}$	$1.591\times 10^{-5}$
F26	$2.340\times 10^{2}$	$1.874\times 10^{-6}$	$1.874\times 10^{-6}$	$1.874\times 10^{-6}$	$2.340\times 10^{2}$	$2.340\times 10^{2}$	$1.874\times 10^{-6}$	$1.874\times 10^{-6}$	$1.874\times 10^{-6}$

**Note:** Corrected *p*-values are calculated as $p_{\mathrm{corrected}}=\min\left(1,p\times 234\right)$, where *p* is the original *p*-value. Values less than 0.05 indicate statistical significance after Bonferroni correction. All statistical analyses were performed using MATLAB R2021a.

**Table 7:** Effect sizes (Cliff’s Delta) for all pairwise comparisons.

Func.	RBMO	PKO	RFO	GA	WOA	GWO	SCA	DE	BKA
F1	−0.400	−0.950	−0.950	−0.950	−0.950	−0.950	−0.950	−0.950	−0.950
F2	−0.350	−0.950	−0.950	−0.950	−0.950	−0.950	−0.950	−0.950	−0.950
F3	−0.300	−0.950	−0.950	−0.950	−0.950	−0.950	−0.950	−0.950	−0.950
F4	−0.450	−0.950	−0.950	−0.950	−0.950	−0.950	−0.950	−0.950	−0.950
F5	−0.380	−0.950	−0.950	−0.950	−0.950	−0.850	−0.950	−0.950	−0.950
F6	−0.200	−0.950	−0.950	−0.950	−0.900	−0.800	−0.950	−0.950	−0.950
F7	−0.100	−0.950	−0.950	−0.900	−0.750	−0.400	−0.800	−0.850	−0.600
F8	−0.150	−0.950	−0.950	−0.950	−0.700	−0.950	−0.950	−0.950	−0.950
F9	−0.420	−0.950	−0.950	−0.950	−0.950	−0.950	−0.950	−0.950	−0.950
F10	−0.180	−0.700	−0.750	−0.950	−0.200	−0.650	−0.950	−0.950	−0.600
F11	−0.410	−0.950	−0.950	−0.950	−0.950	−0.950	−0.950	−0.950	−0.950
F12	−0.100	−0.800	−0.750	−0.950	−0.800	−0.700	−0.950	−0.950	−0.850
F13	−0.430	−0.900	−0.950	−0.950	−0.950	−0.950	−0.950	−0.950	−0.950
F14	−0.360	−0.950	−0.950	−0.950	−0.850	−0.950	−0.950	−0.950	−0.950
F15	−0.340	−0.950	−0.950	−0.950	−0.900	−0.950	−0.950	−0.950	−0.950
F16	0.000	−0.950	−0.950	−0.950	−0.300	−0.950	−0.950	−0.950	−0.950
F17	0.000	−0.950	−0.950	−0.950	0.000	−0.950	−0.950	−0.950	−0.950
F18	0.000	−0.950	−0.950	−0.950	−0.900	−0.950	−0.950	−0.950	−0.950
F19	0.000	−0.950	−0.950	−0.950	0.000	−0.400	−0.950	−0.950	−0.950
F20	−0.340	−0.950	−0.950	−0.950	−0.800	−0.950	−0.950	−0.950	−0.950
F21	−0.180	−0.950	−0.950	−0.900	−0.900	−0.700	−0.850	−0.950	−0.250
F22	−0.170	−0.950	−0.950	−0.750	−0.950	−0.950	−0.600	−0.950	−0.950
F23	−0.120	−0.850	−0.950	−0.750	−0.800	−0.600	−0.800	−0.900	−0.900
F24	0.000	0.000	0.000	−0.950	0.000	−0.950	−0.950	−0.950	−0.950
F25	−0.160	−0.950	−0.950	−0.950	−0.750	−0.950	−0.950	−0.950	−0.950
F26	0.000	−0.950	−0.950	−0.950	0.000	0.000	−0.950	−0.950	−0.950

Note: Negative values indicate that IRBMO performs better than the competing algorithm. Effect size interpretation: |δ|<0.147 (negligible), 0.147≤|δ|<0.33 (small), 0.33≤|δ|<0.474 (medium), |δ|≥0.474 (large).

**Table 8:** Avg performance comparison on selected test functions.

Variant	F1	F2	F8	F9	F14	F15
RBMO (Baseline)	$2.538\times 10^{-3}$	$1.775\times 10^{-2}$	$2.376\times 10^{-10}$	$5.147\times 10^{-4}$	$4.420\times 10^{-7}$	$2.043\times 10^{-2}$
RBMO + a	$7.002\times 10^{-1}$	$2.188\times 10^{1}$	$2.801\times 10^{-6}$	$1.260\times 10^{-1}$	$1.174\times 10^{-6}$	$9.423\times 10^{-118}$
RBMO + a + b	$6.270\times 10^{-4}$	$1.522\times 10^{0}$	$8.717\times 10^{-11}$	$9.035\times 10^{-5}$	$1.769\times 10^{-7}$	$1.473\times 10^{-8}$
RBMO + a + b + c (IRBMO)	$0.000\times 10^{0}$	$0.000\times 10^{0}$	$0.000\times 10^{0}$	$0.000\times 10^{0}$	$0.000\times 10^{0}$	$0.000\times 10^{0}$

**Note:** The best results for each function are highlighted in **bold**.

**Table 9:** Comparison of algorithms on weight minimization of a speed reducer problem.

Algorithm	x1	x2	x3	x4	x5	x6	x7	Result
IRBMO	3.500000	0.700000	17.000000	7.300000	7.715320	3.350541	5.286654	**2994.4245**
RBMO	3.500000	0.700000	17.000000	7.300000	7.715320	3.350541	5.286654	**2994.4245**
PKO	3.500000	0.700000	17.000000	7.300000	7.715320	3.350541	5.286654	**2994.4245**
RFO	3.508880	0.700000	17.000000	7.393655	7.821442	3.358167	5.287076	3003.2866
GA	3.542325	0.701925	17.038054	7.611203	7.998011	3.358032	5.315017	3055.5937
WOA	3.540018	0.700000	17.000000	8.107393	7.862889	3.352130	5.312749	3037.5829
GWO	3.507805	0.700000	17.000000	7.925362	8.034759	3.354718	5.290767	3013.7199
SCA	3.600000	0.700000	17.103570	7.425959	8.300000	3.421596	5.304317	3095.8837
DE	3.569933	0.704929	17.000000	7.874349	8.274293	3.418426	5.290884	3082.5352
BKA	3.502414	0.700000	17.000000	7.300000	7.800952	3.350541	5.287297	2997.6609

**Note:** The best results are highlighted in **bold**.

**Table 10:** Comparison of algorithms on step-cone pulley problem.

Algorithm	d1	d2	d3	d4	w	Result
IRBMO	38.4140	52.8586	70.4727	84.4957	90.0000	**16.0903**
RBMO	38.4234	52.8716	70.4900	84.5164	90.0000	16.1164
PKO	40.8139	56.1636	74.8787	89.7737	84.6993	17.0991
RFO	40.2200	55.3457	73.7884	88.4675	90.0000	$1.7985\times 10^{2}$
GA	39.9103	54.8948	73.1922	87.8073	87.1006	$1.5473\times 10^{7}$
WOA	40.8664	56.2361	74.9749	89.8891	89.7451	$3.5704\times 10^{2}$
GWO	40.9083	56.2969	75.0160	90.0000	86.9167	$1.1263\times 10^{7}$
SCA	42.3934	60.0000	79.9632	90.0000	90.0000	$1.2355\times 10^{11}$
DE	40.4597	57.9465	76.1832	90.0000	90.0000	$9.9335\times 10^{10}$
BKA	40.8094	56.1574	74.8703	89.7637	87.0937	17.6209

**Note:** The best result is highlighted in **bold**.

**Table 11:** Comparison of algorithms on gear train design problem.

Algorithm	x1	x2	x3	x4	Result
IRBMO	38.6093	13.1628	59.0030	59.6984	$0.0000\times 10^{0}$
RBMO	17.7086	12.2256	28.4266	52.7869	$7.3700\times 10^{-27}$
PKO	25.4629	12.0000	35.6138	59.4658	$0.0000\times 10^{0}$
RFO	25.2507	12.4627	47.8165	45.6144	$1.2708\times 10^{-19}$
GA	12.3992	35.3296	54.1513	56.0700	$1.0363\times 10^{-11}$
WOA	12.6469	15.6432	43.9905	31.1707	$0.0000\times 10^{0}$
GWO	42.0324	12.1028	60.0000	58.7641	$2.6724\times 10^{-13}$
SCA	23.4592	15.8104	43.7649	58.7379	$9.8556\times 10^{-12}$
DE	15.8581	12.0000	48.9461	26.9499	$2.5452\times 10^{-10}$
BKA	13.3791	18.0967	34.8548	48.1460	$0.0000\times 10^{0}$

Note: The best results are highlighted in bold.

**Table 12:** Comparison of algorithms on rolling element bearing problem.

Algorithm	Dm	Db	Z	fi	fo	KDmin	KDmax	eps	e	chi	Result
IRBMO	131.2000	18.0000	4.5100	0.6000	0.6000	0.4000	0.6002	0.3000	0.0331	0.6000	**16,958.202**
RBMO	131.2000	18.0000	4.5103	0.6000	0.6000	0.4990	0.6786	0.3000	0.0977	0.6000	**16,958.202**
PKO	131.2000	18.0000	4.6194	0.6000	0.6000	0.4000	0.6000	0.3000	0.1000	0.6000	**16,958.202**
RFO	130.4188	18.0017	4.5831	0.6000	0.6000	0.4894	0.6453	0.3044	0.1000	0.6000	16,974.327
GA	126.3628	18.1893	5.0209	0.5965	0.5948	0.4116	0.6886	0.3206	0.0358	0.6024	17,616.468
WOA	130.8463	18.0001	4.5100	0.6000	0.5397	0.4840	0.7000	0.3087	0.0361	0.6000	17,670.896
GWO	127.9180	18.0033	4.7947	0.6000	0.6000	0.4182	0.6000	0.3410	0.0613	0.6000	17,018.437
SCA	125.0000	18.1390	4.7969	0.6000	0.5830	0.4200	0.6000	0.3036	0.0322	0.6000	17,492.794
DE	127.1235	18.2093	4.9196	0.6000	0.5958	0.4775	0.6929	0.3250	0.0580	0.6000	17,452.190
BKA	131.2000	18.0000	4.5100	0.6000	0.6000	0.4702	0.7000	0.3000	0.0809	0.6000	16,958.251

Note: The best results are highlighted in bold.

## Data Availability

The data presented in this study are available on request from the corresponding author.
